# The teleost fish PepT1-type peptide transporters and their relationships with neutral and charged substrates

**DOI:** 10.3389/fphys.2023.1186475

**Published:** 2023-08-21

**Authors:** Francesca Vacca, Ana S. Gomes, Marco De Gennaro, Ivar Rønnestad, Elena Bossi, Tiziano Verri

**Affiliations:** ^1^ Laboratory of Cellular and Molecular Physiology, Department of Biotechnology and Life Sciences, University of Insubria, Varese, Italy; ^2^ Department of Biological Sciences, University of Bergen, Bergen, Norway; ^3^ Laboratory of Applied Physiology, Department of Biological and Environmental Sciences and Technologies, University of Salento, Lecce, Italy

**Keywords:** SLC15A1, charged amino acids, Atlantic salmon, zebrafish, *Xenopus* oocytes expression, two-electrode voltage clamp

## Abstract

In teleosts, two PepT1-type (Slc15a1) transporters, i.e., PepT1a and PepT1b, are expressed at the intestinal level. They translocate charged di/tripeptides with different efficiency, which depends on the position of the charged amino acid in the peptide and the external pH. The relation between the position of the charged amino acid and the capability of transporting the dipeptide was investigated in the zebrafish and Atlantic salmon PepT1-type transporters. Using selected charged (at physiological pH) dipeptides: i.e., the negatively charged Asp-Gly and Gly-Asp, and the positively charged Lys-Gly and Gly-Lys and Lys-Met and Met-Lys, transport currents and kinetic parameters were collected. The neutral dipeptide Gly-Gln was used as a reference substrate. Atlantic salmon PepT1a and PepT1b transport currents were similar in the presence of Asp-Gly and Gly-Asp, while zebrafish PepT1a elicited currents strongly dependent on the position of Asp in the dipeptide and zebrafish PepT1b elicited small transport currents. For Lys- and Met-containing dipeptides smaller currents compared to Gly-Gln were observed in PepT1a-type transporters. In general, for zebrafish PepT1a the currents elicited by all tested substrates slightly increased with membrane potential and pH. For Atlantic salmon PepT1a, the transport current increased with negative potential but only in the presence of Met-containing dipeptides and in a pH-dependent way. Conversely, large currents were shown for PepT1b for all tested substrates but Gly-Lys in Atlantic salmon. This shows that in Atlantic salmon PepT1b for Lys-containing substrates the position of the charged dipeptides carrying the Lys residue defines the current amplitudes, with larger currents observed for Lys in the N-terminal position. Our results add information on the ability of PepT1 to transport charged amino acids and show species-specificity in the kinetic behavior of PepT1-type proteins. They also suggest the importance of the proximity of the substrate binding site of residues such as Lys^PepT1a^/Gln^PepT1b^ for recognition and specificity of the charged dipeptide and point out the role of the comparative approach that exploits the natural protein variants to understand the structure and functions of membrane transporters.

## 1 Introduction

Peptide transporter 1 (PepT1), also named member 1 of the solute carrier family 15 (Slc15a1), is known for mediating the uptake of di/tripeptides in the intestine of vertebrates (Daniel, 2004). In teleosts, two PepT1-type transporters are present, i.e., PepT1a and PepT1b, both expressed at the intestinal level, but also in other organs/tissues [see, e.g., Vacca et al. (2019) for the general organ/tissue distribution of PepT1a (slc15a1a) and PepT1b (slc15a1b) mRNA in teleosts, and (Gomes et al., 2020) for a detailed spatial and tissue distribution of Atlantic salmon (*Salmo salar*) peptide transporter 1a (PepT1a) and 1b (PepT1b)]. Their function has been analyzed *in vitro* in Atlantic salmon [see, e.g., (Rønnestad et al., 2010; Gomes et al., 2020), zebrafish (*Danio rerio*)] [see, e.g., (Verri et al., 2003; Vacca et al., 2019)], and European seabass (*Dicentrarchus labrax*) [see, e.g., (Sangaletti et al., 2009)]. The regulation of their expression by environmental factors has also been studied, e.g., in mummichog (*Fundulus heteroclitus*) [see, e.g., (Bucking and Schulte, 2012)], Nile (*Oreochromis niloticus*) and Mozambique (*Oreochromis mossambicus*) tilapia [see, e.g., (Huang et al., 2015; Orozco et al., 2017; Chourasia et al., 2018; Hallali et al., 2018; Con et al., 2019)] and European seabass [see, e.g., (Rimoldi et al., 2015)].

In fish, the molecular basis for the recognition of neutral vs. charged peptides by PepT1a and PepT1b proteins remains still elusive. In mammals, the binding domain of PepT1 is asymmetric and the transport of cationic dipeptides depends on the location of the charged side chain within the substrate molecule. Notably, when the charged residue is provided in the N-terminal position, the substrate is transported in both zwitterionic and positively charged forms, but if the charged residue is provided in the C-terminal position, only the neutral form is transported. On the other hand, anionic dipeptides are mainly transported in their neutral form, with only a minimal fraction being transiently protonated in the proximity of the cellular membrane and, thus, translocated virtually as charged dipeptides (Kottra et al., 2002). The transport of charged dipeptides in PepT1b was first shown in zebrafish (Verri et al., 2010) and then in zebrafish and seabass (Margheritis et al., 2013). Data on the kinetic parameters in the presence of Lys- (Lys-Gly and Gly-Lys) and Asp-containing (Asp-Gly and Gly-Asp) dipeptides did demonstrate that the position of the charged side chain within the dipeptide also affects the piscine PepT1 transport process (Verri et al., 2010). Further studies focused on the transport kinetics of substrates made of Lys and/or Met due to the importance of such amino acids in animal nutrition. In fact, these are essential amino acids known to impact body metabolism and to be crucial for growth performances in vertebrates, particularly in teleost fish (Verri et al., 2017). This is relevant when, e.g., plant-based protein sources are used for feeding farmed carnivorous fish as a replacement for fish meal (Kaushik and Seiliez, 2010). In fact, plant proteins are limited in essential amino acids, such as Lys, Met, Arg, and Leu; thus, supplementation of such amino acids in diets as per the requirement of fish is often necessary to avoid reduced growth and potential health issues in carnivorous fish (El-Husseiny et al., 2017; Kousoulaki et al., 2021).

Early and later studies had already shown that a piscine PepT1-type transporter such as zebrafish PepT1b can strongly diverge in function from its mammalian and fish counterparts (Sangaletti et al., 2009; Rønnestad et al., 2010; Margheritis et al., 2013; Verri et al., 2017), this suggesting that the molecular diversity among PepT1-type proteins might have been at the basis of the functional differences observed. Indeed, Margheritis et al. (2013) had also identified that the amino acid substitution Ile334Thr in the zebrafish PepT1b transmembrane domain 8 is relevant for justifying the functional differences observed in the zebrafish PepT1b with respect to the rabbit and seabass transporters. Therefore, investigating functional aspects in the “natural variants” of PepT1-type proteins might be important to deeply understand their role in animal physiology. In this work, we exploit the endogenous differences in piscine PepT1a and PepT1b proteins for understanding the transport of (four) positively and (two) negatively charged dipeptides at two different pH, suggesting molecular determinants involved in the interaction of the ligands on the proteins, taking also into account the structural models of PepT1 recently proposed [see, e.g., (Killer et al., 2021), and literature cited therein].

## 2 Materials and methods

### 2.1 Structure comparisons

#### 2.1.1 Software and tools

The following software/tools were used in this work: AutoDock Vina (Trott and Olson, 2010; Eberhardt et al., 2021), SWISS-MODEL (Bienert et al., 2016), UCSF ChimeraX (Pettersen et al., 2004), OpenBabel (O'Boyle et al., 2011), MGLTools 1.5.6 (Morris et al., 2009), PyMOL (Schrödinger and DeLano, 2020), ChemAxon (https://chemaxon.com/) and BIOVIA Discovery Studio Visualizer (BIOVIA, Dassault Systèmes, Discovery Studio, v. 21.1.0.20298, San Diego: Dassault Systèmes, 2021).

#### 2.1.2 Protein preparation for docking analysis

Based on a preliminary set of simulations on structures of human (*Homo sapiens*) transporter PepT1 [HsPepT1; (Killer et al., 2021)] (for details, see Supplementary Methods and Supplementary Figure S1; Supplementary Table S1 within), molecular docking analyses were performed using i) zebrafish PepT1a (zfPepT1a) and PepT1b (zfPepT1b) and Atlantic salmon PepT1a (asPepT1a) and PepT1b (asPepT1b) protein models (target proteins) and ii) Ala-Phe, Gly-Gln, Gly-Asp, Asp-Gly, Gly-Lys, Lys-Gly, Met-Lys, Lys-Met (tested dipeptides). In particular, using SWISS-MODEL and starting from their primary sequences, i.e., Acc. No. QHB80166.1 (zfPepT1a), Acc. No. NP_932330.1 (zfPepT1b), Acc. No. XP_014028426.2 (asPepT1a), Acc. No. NP_001140154.1 (asPepT1b), the four teleost fish target proteins were modelled on the apoprotein (apo) HsPepT1 in the outward facing open conformation and on HsPepT1 bound to Ala-Phe in the outward facing open conformation (PDB id: 7PN1 and PDB id: 7PMX, respectively).

### 2.2 Expression in *Xenopus laevis* oocytes and electrophysiology

The complete open reading frame encoding for Atlantic salmon PepT1a (Gomes et al., 2020) and PepT1b (Rønnestad et al., 2010), zebrafish PepT1a (Vacca et al., 2019) and PepT1b (Verri et al., 2003) were subcloned into pSPORT1 plasmid. *Xenopus laevis* oocytes were collected under anaesthesia [MS222; 0.10% (w/v) solution in tap water] by laparotomy from adult females and prepared as described in (Bhatt et al., 2022). *Xenopus laevis* frogs were maintained according to international guidelines (Delpire et al., 2011; McNamara et al., 2018). The animal study was reviewed and approved by the Committee of the “Organismo Preposto al Benessere degli Animali” of the University of Insubria and nationally by Ministero della Salute (permit nr. 449/2021-PR). Capped cRNAs were synthesized by *in vitro* transcription using T7 RNA polymerase from cDNAs in pSPORT1 linearized with NotI and purified with Wizard SV Gel and PCR clean-up system (Promega Italia, Milan, Italy). The purified cRNAs were quantified using a NanoDropTM 2000 Spectrophotometer (Thermo Fisher Scientific, Monza, Italy), and 25 ng was injected into the oocytes using a manual microinjection system (Drummond Scientific Company, Broomall, PA, United States). Before electrophysiological studies, the cRNA-injected oocytes were incubated at 18°C for 3–4 days in NDE (NaCl 96 mmol/L, KCl 2 mmol/L, CaCl_2_ 1.8 mmol/L, MgCl_2_ 1 mmol/L, HEPES 5 mmol/L, pyruvate 2.5 mmol/L and gentamycin sulphate 0.05 mg/mL pH 7.6). Two-electrode voltage-clamp experiments were performed using a commercial amplifier (Oocyte Clamp OC-725B, Warner Instruments, Hamden, CT, United States) and the pCLAMP software (Version 10.7, Molecular Devices, San Jose CA Sunnyvale, CA, United States). The holding potential was kept at −60 mV; the voltage pulse protocol consisted of 10 square pulses from −140 to +20 mV (20 mV increment) of 400 ms each. Signals were filtered at 0.1 kHz, sampled at 0.2 kHz or 0.5 kHz, and 1 kHz. Transport-associated currents (*I*_tr_) were calculated by subtracting the traces in the absence of substrate from those in its presence. The values of the steady-state currents were normalized to the value of the current recorded for each transporter at −140 mV in the presence of 1 mmol/L Gly-Gln at pH 7.6. The number of samples *n* corresponds to the number of oocytes used in each condition and the batches correspond to the animals from which the oocytes were collected.

### 2.3 Data analysis

Steady-state transport currents from substrate dose-response experiments were fitted with the Michaelis-Menten equation [1]:

$$\left[I_{0}=\frac{-I_{\max}}{1+\left(\frac{\left[S\right]}{K_{0.5}}\right)}+I_{\max}\right]$$ (1)

for which *I*_0_ is the evoked current, *I*_max_ is the derived relative maximal current, *S* is the substrate concentration and *K*_0.5_ is the substrate concentration at which current is half-maximal. Data were analyzed using Clampfit 10.7 (Molecular Devices). All figures and statistics (Descriptive Statistic; Shapiro-Wilk Normality test; Two sample *t*-test and Mann-Whitney Test) were generated with Origin 8.0 (OriginLab, Northampton, MA, United States). Details of statistical analysis and all the results of the tested samples are reported in Statistical Summary Document Supplementary Tables S2–S4.

### 2.4 Solutions

The external control solution had the following composition: NaCl 98 mmol/L, MgCl_2_ 1 mmol/L, CaCl_2_ 1.8 mmol/L. For pH 5.5 and 6.5 the buffer solution Pipes 5 mmol/L was used, while for pH 7.6 and 8.5 Hepes 5 mmol/L. Final pH values were adjusted with HCl or NaOH. The substrate dipeptides tested were: Gly-Gln, Gly-Asp, Asp-Gly (Sigma-Aldrich-Merck-Italy, Milan) and Gly-Lys, Lys-Gly, Met-Lys, Lys-Met (Genicbio-China, Shanghai). All tested substrates were added at the indicated concentrations (from 3 µmol/L to 30 mmol/L) in the external solutions with the appropriate pH. For each tested dipeptide, the percentage of positively, negatively and/or zwitterionic microspecies present at pH 5.5, 6.5, 7.6, and 8.5 is reported in the Supplementary Methods (see Supplementary Table S1).

## 3 Results

### 3.1 Three-dimensional structures of the Atlantic salmon and zebrafish PepT1-type transporters

**FIGURE 1:**
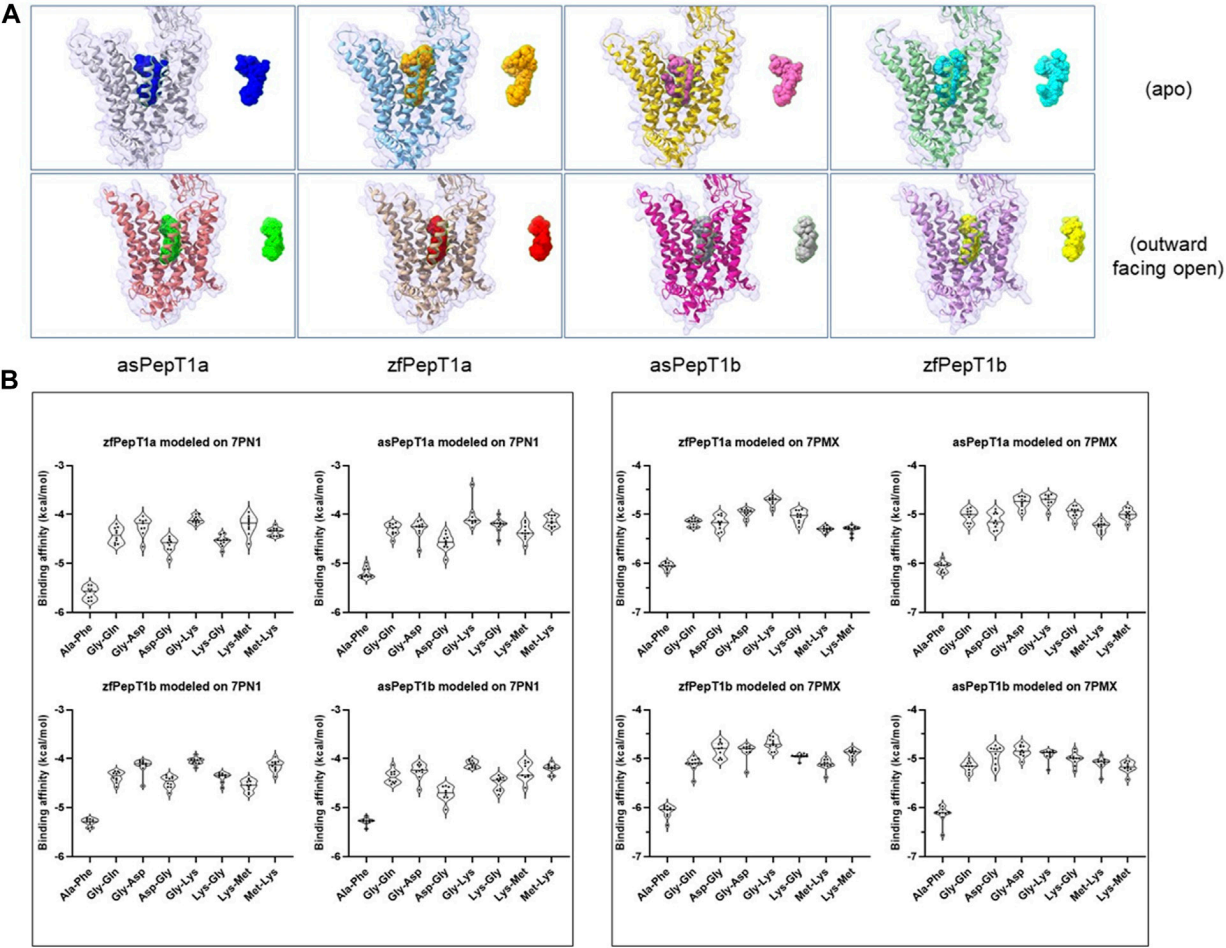
**(A)** ChimeraX analysis of protein-ligand complexes as obtained by molecular docking simulation performed using Ala-Phe, Gly-Gln, Gly-Asp, Asp-Gly, Gly-Lys, Lys-Gly, Met-Lys, Lys-Met (tested dipeptides) and zebrafish PepT1a (zfPepT1a) and PepT1b (zfPepT1b) and Atlantic salmon PepT1a (asPepT1a) and PepT1b (asPepT1b) protein models (target proteins), representing two structural conformations [i.e., the apoprotein (Apo) in the outward facing open conformation, 7PN1 (apo) and the protein bound to the peptide in the outward facing open conformation, 7PMX (outward facing open)]. For sake of clarity, the figure shows the superimposed positions of all the ligands after the calculation of the lower binding energy states in the molecular docking simulation (see also Supplementary Methods and Supplementary Figure S1 for details). **(B)** AutoDock Vina results of the binding affinity (expressed in Kcal/mol) for each predicted ligand-protein complex before (left) and after (right) using the “Protonation” feature online tool available in ChemAxon to calculate pK_a_ and the protonation state of each dipeptide molecule at pH 7.5 (for details, see Supplementary Methods, paragraph 1.3 Ligands preparations). Lower energy values indicate a better stability of the ligand-protein complex.

We ascertained the possibility to use the 3D model of *Homo sapiens* PepT1 (HsPepT1) either not bound (apo; PBD id: 7PN1) or bound to Ala-Phe in the outward facing open conformation (outward facing open; PDB id: 7PMX) to study our fish transporters for their physiological interactions with selected neutral, positively and negatively dipeptides (namely, Ala-Phe, Gly-Gln, Gly-Asp, Asp-Gly, Gly-Lys, Lys-Gly, Met-Lys, Lys-Met) (see Supplementary Figure S1). Then, we generated (a set of) molecular docking structures of zfPepT1a, asPepT1a, zfPepT1b, and asPepT1b. After modelling, the predicted structures of the four transporters were subjected to a conversion of the PDB files in “pdbqt” digital format files, as required to run Autodock Vina. The preparation of ligands and the molecular docking simulation protocol are detailed in Supplemental Methods. The results are summarized in Figure 1. By comparing the set of “apo” structures with those in the “outward facing open” configuration it clearly emerges that the “apo” conformation invariably allocates the various substrates in a more flexible way than the “outward facing open” conformation (please compare the superimposed substrates in the upper line with those depicted in the lower line of structures in Figure 1A). In fact, the surface of contact interaction of the “apo” structures is generally larger than that of the “outward facing open” conformation, the latter representing in the database the structure bound to the substrate (i.e., Ala-Phe). Moreover, while the “outward open facing” contact surface appears more similar in the four structures, each “apo” structure seems to offer a unique contact surface interaction to substrates, which suggests that each zebrafish and Atlantic salmon transporter may differently interact with the dipeptide substrates from the outer (extracellular) part of the protein, thus exhibiting a sort of plasticity in the early phases of substrate recognition. Likewise, a larger interaction surface means that the substrate dipeptides can virtually interact with a larger number of residues of the transporter, and thus have different (options of) sites of interaction. That different substrates do interact differently with the proteins in the various conformations is confirmed by the binding energies reported in Figure 1B.

### 3.2 Characterization of the transport of charged substrates

#### 3.2.1 Transport currents of positively and negatively charged dipeptides

**FIGURE 2:**
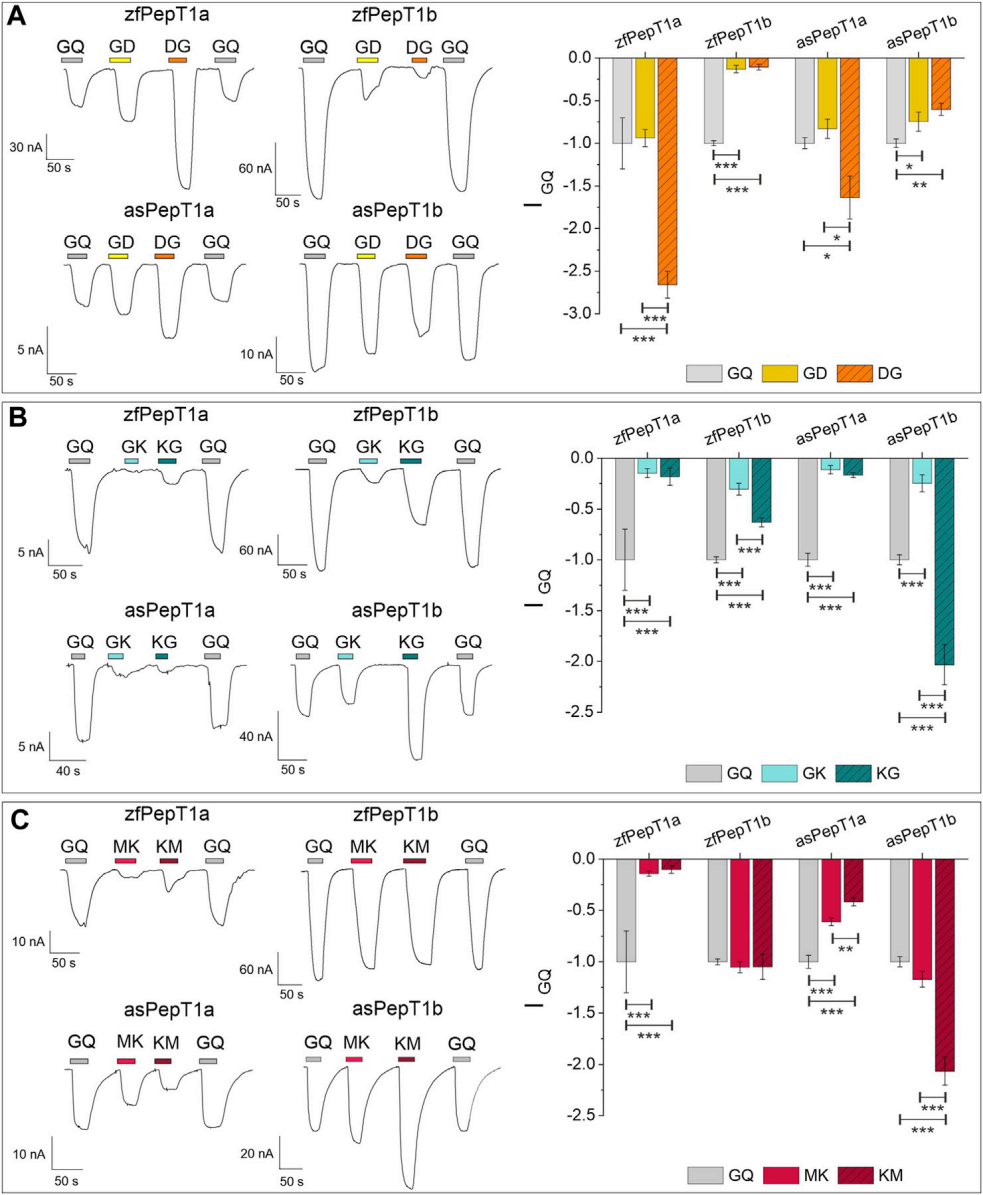
Transport current elicited by charged dipeptides compared to Gly-Gln (GQ) (all at 1 mmol/L) in zebrafish PepT1a and PepT1b (zfPepT1a, zfPepT1b) and Atlantic salmon PepT1a and PepT1b (asPepT1a and asPepT1b). The transport current elicited by Gly-Asp and Asp-Gly (GD, DG) in **(A)**, by Gly-Lys and Lys-Gly (GK, KG) in **(B)**, by Met-Lys and Lys-Met (MK, KM) in **(C)** were recorded at the holding potential of −60 mV and at pH 7.6. In the left part of all figures, representative traces of transport current induced by each substrate are indicated by the different colored bars. In the right part of **(A,B,C)** the mean of the transport-associated current was normalized to Gly-Gln current. Values are mean ± SE from 7 to 15 oocytes from different batches. The statistical details (Experimental Question, Statistical Test used, *n* of sample, *p* value, etc.) are reported in Statistical Summary Document Supplementary Table S2.

A comparative analysis of PepT1a and PepT1b transport-associated currents in the presence of charged peptides was performed. To assess the relevance of the position of a positively or negatively charged residue in the interaction with the PepT1a and PepT1b proteins, experiments were conducted in the presence of dipeptides with a charged amino acid in either the N- or C-terminal position, at pH 7.6. The data recorded are summarized in Figure 2A for the negatively charged (anionic) dipeptides Gly-Asp and Asp-Gly, and in Figures 2B, C for the positively charged (cationic) substrates Gly-Lys and Lys-Gly and Met-Lys and Lys-Met. The representative traces recorded at −60 mV were normalized to the current elicited by 1 mmol/L of the reference (neutral) substrate Gly-Gln. In the representative traces and the histogram reported in Figure 2A, Asp-Gly evokes significative large currents in PepT1a-expressing oocytes if compared to Gly-Gln (see Statistical Summary Document Supplementary Table S2 for the statistical detail). Changing the position of the negatively charged residue, i.e., with Gly-Asp, the amplitude of the transport-associated current decreases in PepT1a transporters, becoming not significantly different to that generated by Gly-Gln. The currents evoked by the two anionic substrates are comparable and the current values are smaller than Gly-Gln in Atlantic salmon PepT1b and greatly reduced in zebrafish PepT1b, as visible in the representative traces. The statistical comparison confirmed the differences (see Statistical Summary Document Supplementary Table S2 for the statistical detail). Thus, the position of the negatively charged residue in the dipeptide does not appear to be a discriminating factor for the transport activity of PepT1b.

In the presence of dipeptides containing the cationic amino acid Lys, the transport currents evoked in both PepT1a transporters are always smaller than Gly-Gln, regardless of the position of the positively charged residue in the dipeptide, as visible in the representative traces (Gly-Lys and Lys-Gly in Figure 2B; Met-Lys and Lys-Met in Figure 2C). Only in Atlantic salmon PepT1b, Lys-Gly and Lys-Met elicit a mean current amplitude larger than that of the reference substrate Gly-Gln (Figures 2B, C). For zebrafish PepT1b Gly-Lys and Lys-Gly currents are significantly reduced in amplitude if compared to those generated by Gly-Gln. Comparing the currents evoked by Gly-Lys and Lys-Gly, the latter evokes always larger currents (Figure 2B). The differences are statistically significant in all the transporters. When the neutral residue Gly is substituted by Met, an essential amino acid, the importance of the charge position is evident in Atlantic salmon PepT1b (Figure 2C), since the current evoked by Lys-Met is significantly higher than that in the presence of Met-Lys, whereas in zebrafish PepT1b Lys-Met and Met-Lys show comparable currents that are not statistically different from the current elicited by Gly-Gln (Figure 2C).

#### 3.2.2 Voltage dependence of the transport of charged dipeptides

**FIGURE 3:**
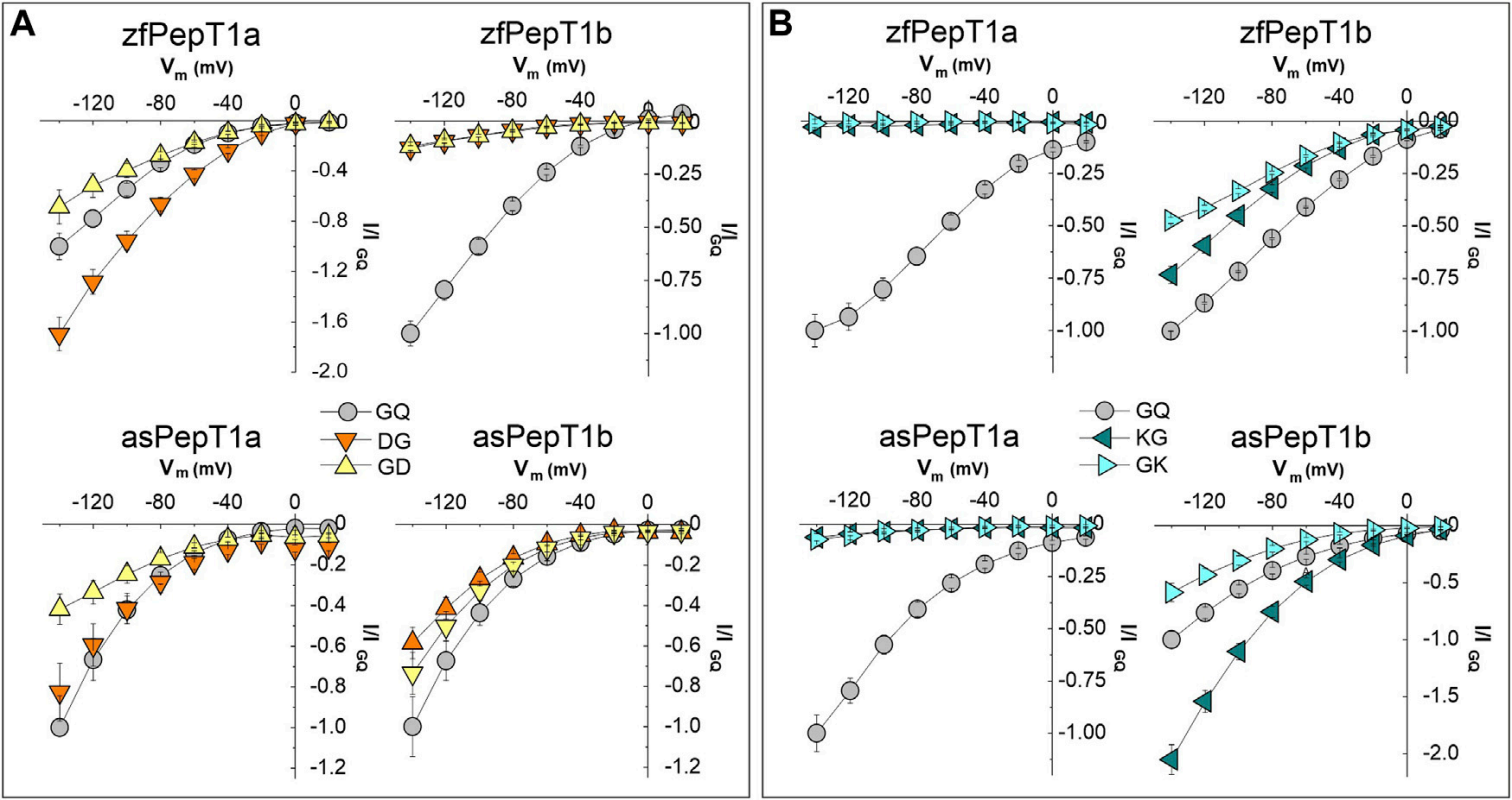
Current-voltage (*I*/*V*) relationships of transport-associated currents in the presence of 1 mmol/L of Asp- and Lys-containing dipeptides in zebrafish (zfPepT1a and zfPepT1b, top) and Atlantic salmon (asPepT1a and asPepT1b, bottom) transporters. **(A)**, *I*/*V* in the presence of Asp-Gly (DG, orange-down triangles), Gly-Asp (GD, yellow-up triangles) and Gly-Gln (GQ, gray circle) at pH 7.6; **(B)**, *I*/*V* in the presence of Lys-Gly (KG, dark cyan-left triangles), Gly-Lys (GK, light cyan-right triangles) and Gly-Gln (GQ, gray-circle) at pH 6.5. The currents elicited by voltage pulses in the range from −140 to +20 mV (20 mV steps from *V*_h_ of −60 mV) are obtained by subtracting from the current in the presence of substrate the current in its absence, and are presented as mean ± SE from 7 to 15 oocytes from different batches normalized with respect to the current value of Gly-Gln at −140 mV. The statistical details (Experimental Question, Statistical Test used, *n* of sample, *p* value, etc.) are reported in Statistical Summary Document Supplementary Table S3.

To assess how membrane voltage affects the transport of the charged dipeptides, the transporters were tested in the presence of 1 mmol/L of anionic (Asp-Gly and Gly-Asp) and cationic (Lys-Gly and Gly-Lys) dipeptides by applying the pulse protocol (step of 20 mV from −140 to +20 mV). The transport currents of anionic substrates were recorded at pH 7.6 while the currents of cationic substrates at pH 6.5 to have about 98% of the molecules in the charged form as for the Henderson-Hasselbalch equation (see Supplementary Table S1 for details). For all substrates, the currents were normalized to the Gly-Gln current value at the membrane potential of −140 mV and the *I*/*V* relationships then reported in Figures 3A, B. All but zebrafish PepT1b show voltage-dependent currents in the presence of both Asp-Gly and Gly-Asp with an increment of the inward currents recorded from +20 mV to −140 mV (Figure 3A). According to the current values at −60 mV, Asp-Gly evokes a statistically significant higher current than Gly-Gln at all tested potentials in zebrafish PepT1a, but not in Atlantic salmon PepT1a, in which the *I*/*V* relationships are similar for Asp-Gly and Gly-Gln (statistical analysis is reported in Statistical Summary Document Supplementary Table S3). For both PepT1a transporters, the comparison of the *I*/*V* curve of Gly-Asp with Gly-Gln shows a similar behavior from −20 mV to −80 mV. A decrease in Gly-Asp current amplitude compared to Gly-Gln is noted at hyperpolarization conditions, from −100 mV to −140 mV. In PepT1b transporters, the *I*/*V* relationships are similar for the anionic dipeptides, with the currents invariably smaller than those elicited by Gly-Gln. In Atlantic salmon PepT1b, an increase of Asp-Gly and Gly-Asp currents at the most hyperpolarizing membrane potentials is observed.

At all tested potentials, the transport of cationic dipeptides does not elicit considerable currents in both PepT1a transporters. Conversely, species-specific differences are observed by comparing the *I*/*V* curves of the PepT1b transporters in the presence of cationic dipeptides, especially Lys-Gly (Figure 3B). Lys-Gly is the best substrate at all tested potentials if compared to the reference substrate Gly-Gln in Atlantic salmon PepT1b (see Statistical Summary Document Supplementary Table S3), while in zebrafish smaller currents than those of Gly-Gln are observed. For both PepT1b transporters and at all tested potentials, Gly-Lys elicits smaller currents with respect to those due to Gly-Gln. Moreover, in zebrafish PepT1b, the position of the charged residue in the dipeptide influences the voltage-dependence of the current amplitude in the range from −80 to −140 mV with a larger current recorded in the presence of Lys-Gly than Gly-Lys. To fully characterize the PepT1a and PepT1b proteins and their role in transporting charges, important nutritional peptides such as Lys- and Met-containing dipeptides were tested. The pulse protocol at two different pH conditions, i.e., 7.6 and 6.5, used for investigating membrane voltage and external pH dependence of the transport currents (see also the next paragraph), respects the same experimental conditions reported in Margheritis et al. (2013).

#### 3.2.3 pH dependence of the transport of positively charged dipeptides

**FIGURE 4:**
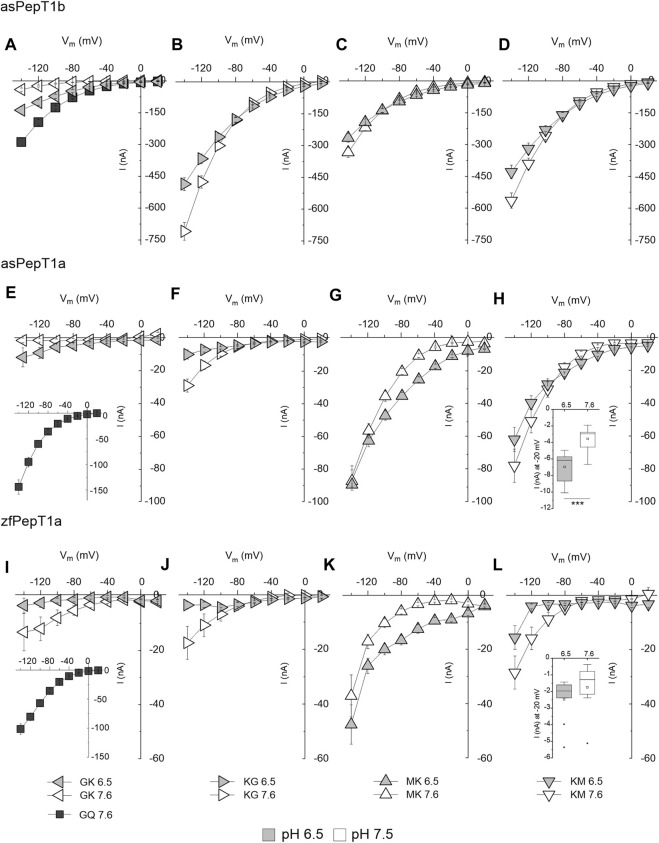
Current-voltage (*I*/*V*) relationships of transport-associated currents in the presence of lysine-containing peptides in Atlantic salmon PepT1b (asPepT1b) in **(A–D)**, Atlantic salmon PepT1a (asPepT1a) in **(E–H)** and zebrafish PepT1a in **(I–L)** (zfPepT1a). The currents elicited by voltage pulses in the range −140 to +20 mV (20 mV steps from *V*_h_ of −60 mV) were recorded in the presence of 1 mmol/L of different substrates [Gly-Lys (GK) in **(A,E,I)**; Lys-Gly (KG) in **(B,F,J)**; Met-Lys (MK) in **(C,G,K)**; Lys-Met (KM) in **(D,H,L)**] at pH 6.5 (light grey symbols) and pH 7.6 (empty symbols). Gly-Gln (GQ) values at pH 7.6 are reported as reference (dark grey symbols). The current values reported in the *I*/*V* relationship were the obtained by subtracting from the current in the presence of the substrate the current in its absence. Data are reported as mean ± SE from 8 to 11 oocytes from different batches. The statistical details (Experimental Question, Statistical Test used, *n* of sample, *p* value, etc.) are reported in Statistical Summary Document Supplementary Table S4.

Current-voltage relationships are reported for zebrafish PepT1a and Atlantic salmon PepT1a and PepT1b transporters at pH 6.5 and 7.6 (Figure 4). In Atlantic salmon PepT1b, the current generated by Gly-Lys at pH 6.5 is significantly higher (*p* < 0.001) in the potential range from −20 mV to −140 mV (Figure 4A). The currents evoked by Lys-Gly, Met-Lys, and Lys-Met depend on the external pH as a function of the membrane potential, with significant differences in current amplitude at −20 mV: *p* < 0.001 for Lys-Gly and Met-Lys and *p* < 0.05 for Lys-Met. At the most negative voltages (−120 mV and −140 mV), the currents increase in the presence of the same substrates changing from pH 6.5 to 7.6, with significantly different values at −140 mV: *p* < 0.001 for Lys-Gly and *p* < 0.05 for Met-Lys and Lys-Met (Figures 4B–D). In PepT1a transporters, no effect of pH is observable in Gly-Lys and Lys-Gly induced currents (see Figures 4I, J for zebrafish transporter, Figures 4E, F for Atlantic salmon PepT1a). The only exception is Lys-Gly, which generates a current significantly higher at pH 7.6 at −140 mV: *p* < 0.01 for Atlantic salmon PepT1a and *p* < 0.05 for zebrafish PepT1a (Figures 4F, J). Concerning Met-Lys, the effect of pH on the current-voltage relationship is similar in both PepT1a transporters. The transport currents are significantly higher at pH 6.5 in the range from −20 mV to −100 mV with *p* < 0.001 at −20 mV and *p* < 0.01 at −100 mV for Atlantic salmon (Figure 4G). For zebrafish, the transport currents are significantly higher at pH 6.5 in the range varying from −20 mV to −120 mV with *p* < 0.001 at −20 mV and *p* < 0.05 at −120 mV (Figure 4K). In the voltage range from −140 mV to −60 mV, the current evoked by Lys-Met apparently increases but without any statistically significant difference with respect to the current recorded at pH 6.5 for both PepT1a (Figures 4H, L). Once set the membrane voltage at −20 mV, the same substrate shows currents higher at pH 6.5 than at pH 7.6 with significant differences for Atlantic salmon PepT1a (*p* < 0.001 at −20 mV); however, no statistically significant differences emerge for the zebrafish transporter. The current value at −20 mV cannot be appreciated by the data reported in the *I*/*V* relationship; thus, for this membrane voltage values the data are reported in the insets (Figures 4H, L) (see Statistical Summary Document Supplementary Table S4 for details).

#### 3.2.4 Substrate affinity

The transport currents for Lys-Gly at increasing concentrations from 0.1 to 30 mmol/L, were collected by perfusing the oocytes expressing Atlantic salmon PepT1a and PepT1b at pH values of 7.6 at the holding potential of −60 mV.

**FIGURE 5:**
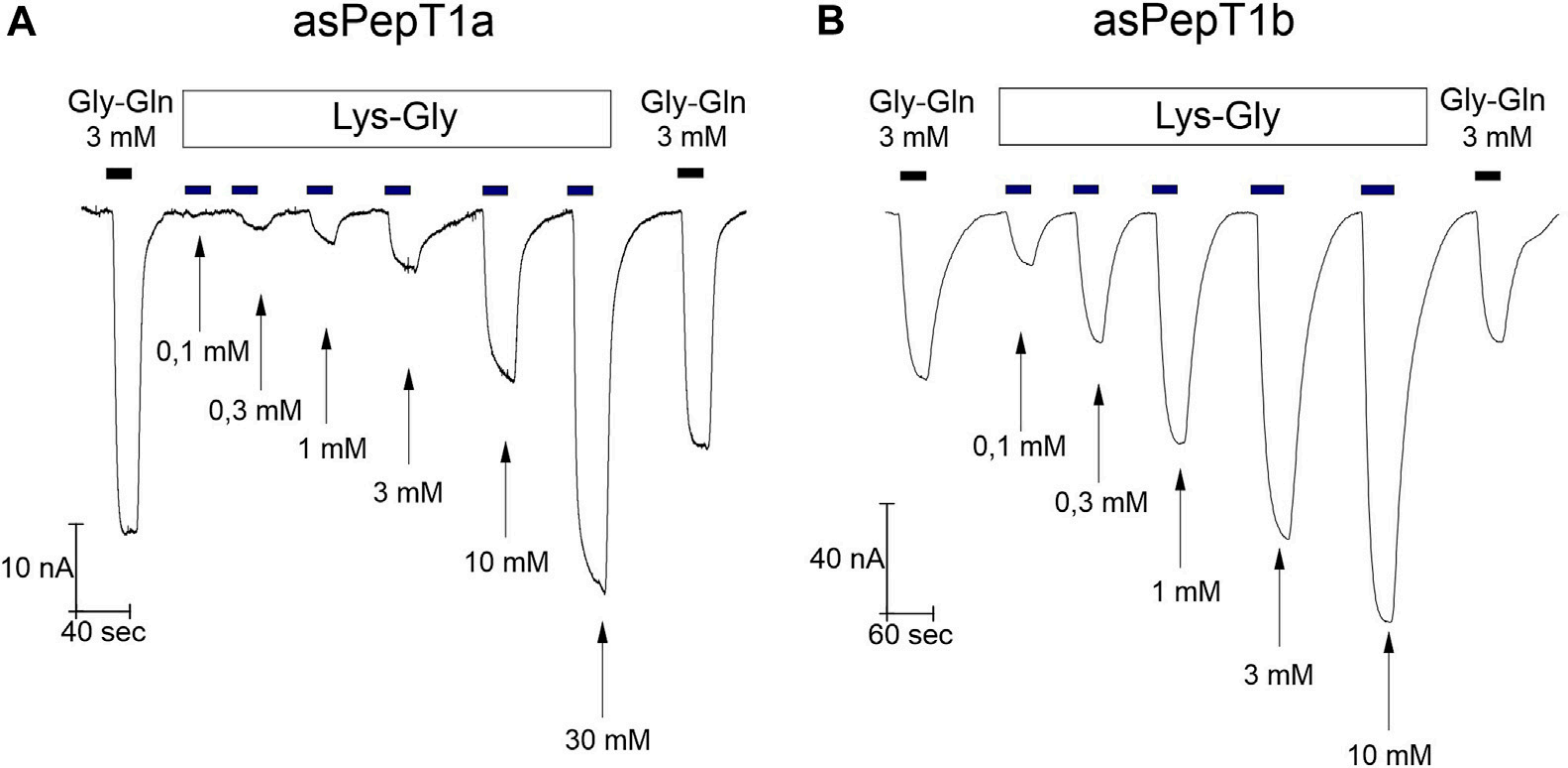
Currents in *Xenopus laevis* oocytes expressing Atlantic salmon PepT1a (asPepT1a) in **(A)**, and Atlantic salmon PepT1b (asPepT1b) in **(B)**. The currents were recorded at the holding potential (*V*_h_) of −60 mV at pH 7.6. The oocytes were perfused consecutively with increasing concentrations of Lys-Gly from 0.1 mmol/L to 10 mmol/L for PepT1b and to 30 mmol/L for PepT1a. The top bars indicate the substrate administration, and the bottom arrows indicate the different concentrations of substrate. The current of reference substrate Gly-Gln at 3 mmol/L was recorded at the start and at the end of each trace, as indicated by the top black bars.

**FIGURE 6:**
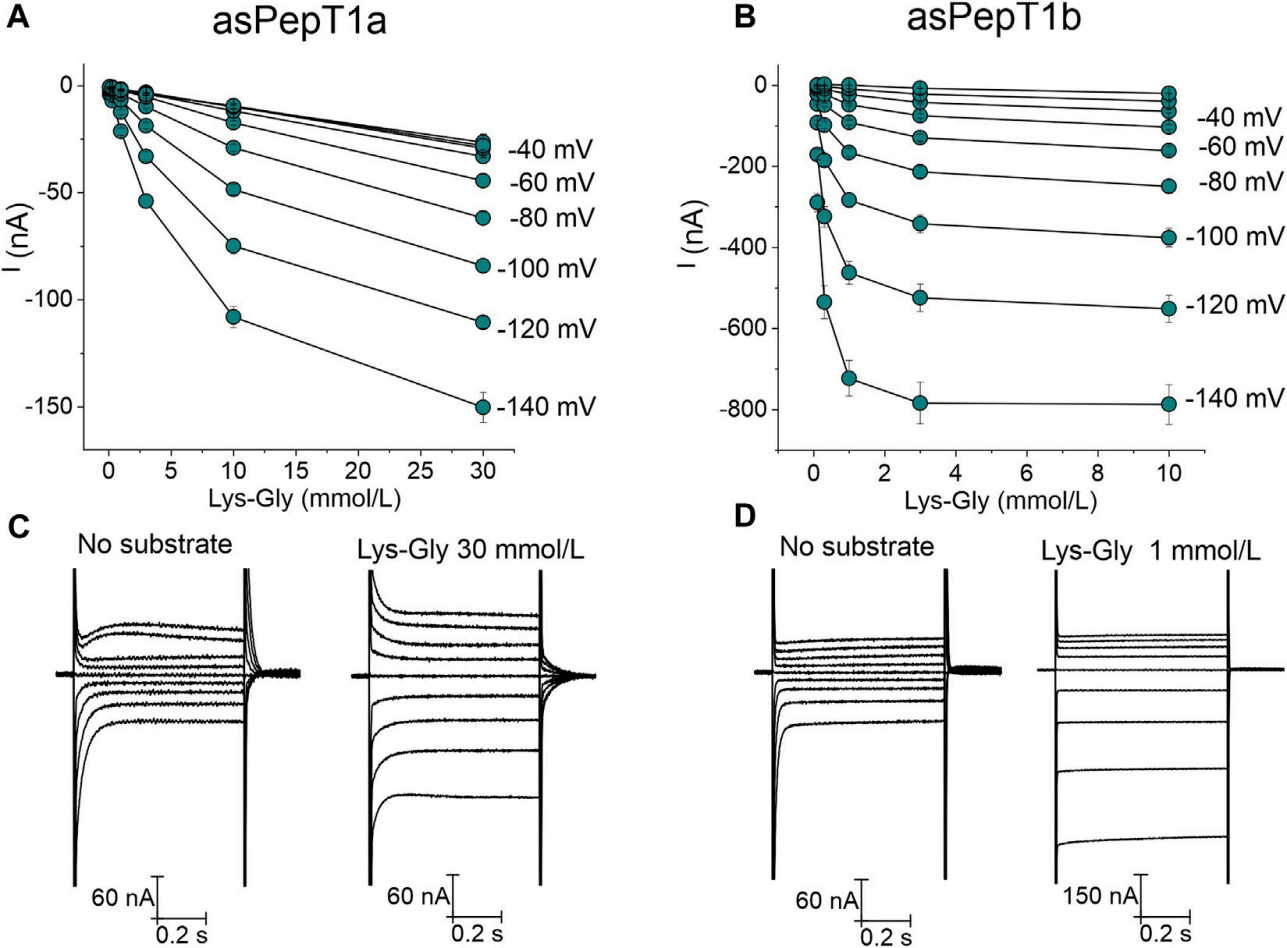
Current-substrate concentration relationships at different voltage conditions in Atlantic salmon PepT1a in **(A)** and PepT1b in **(B)**. The current values at each tested concentration of Lys-Gly (from 0.1 mmol/L to 10 mmol/L for PepT1b and to 30 mmol/L for PepT1a) were obtained by subtracting the current traces in the absence of substrate from those occurring in its presence. Data are reported as mean ± SE of 10 or 12 oocytes from 3 batches. In **(C,D)**, respectively: the representative traces of Atlantic salmon PepT1a and PepT1b in the absence of substrate [in the left part of **(C,D)**] and in the presence of 30 mmol/L of Lys-Gly for PepT1a [in the right part of **(C)**] and in the presence of 1 mmol/L of Lys-Gly for PepT1b [in the right part of **(D)**].

Increasing Lys-Gly concentration enhanced the transport current (Figure 5) for Atlantic salmon PepT1a and PepT1b. But, at the same concentration of 1 mmol/L Lys-Gly gave rise only to small currents in PepT1a expressing oocytes (Figure 5A) compared to those of PepT1b (Figure 5B). When the same concentrations of Lys-Gly were tested from −140 mV to 0 mV, the current*-*concentration relationships showed that for PepT1a the maximal current was never reached at the tested voltages (Figure 6A). Moreover, in the presence of the Lys-Gly 30 mmol/L, Atlantic salmon PepT1a showed residual pre-steady state currents (Figure 6C). Conversely, Atlantic salmon PepT1b currents in the presence of 1 mmol/L Lys-Gly were like those recorded at higher substrate concentrations at all tested potentials (Figure 6B). The absence of pre-steady state currents in the presence of 1 mmol/L Lys-Gly confirms the saturation of the transport in PepT1b (Figure 6D).

**FIGURE 7:**
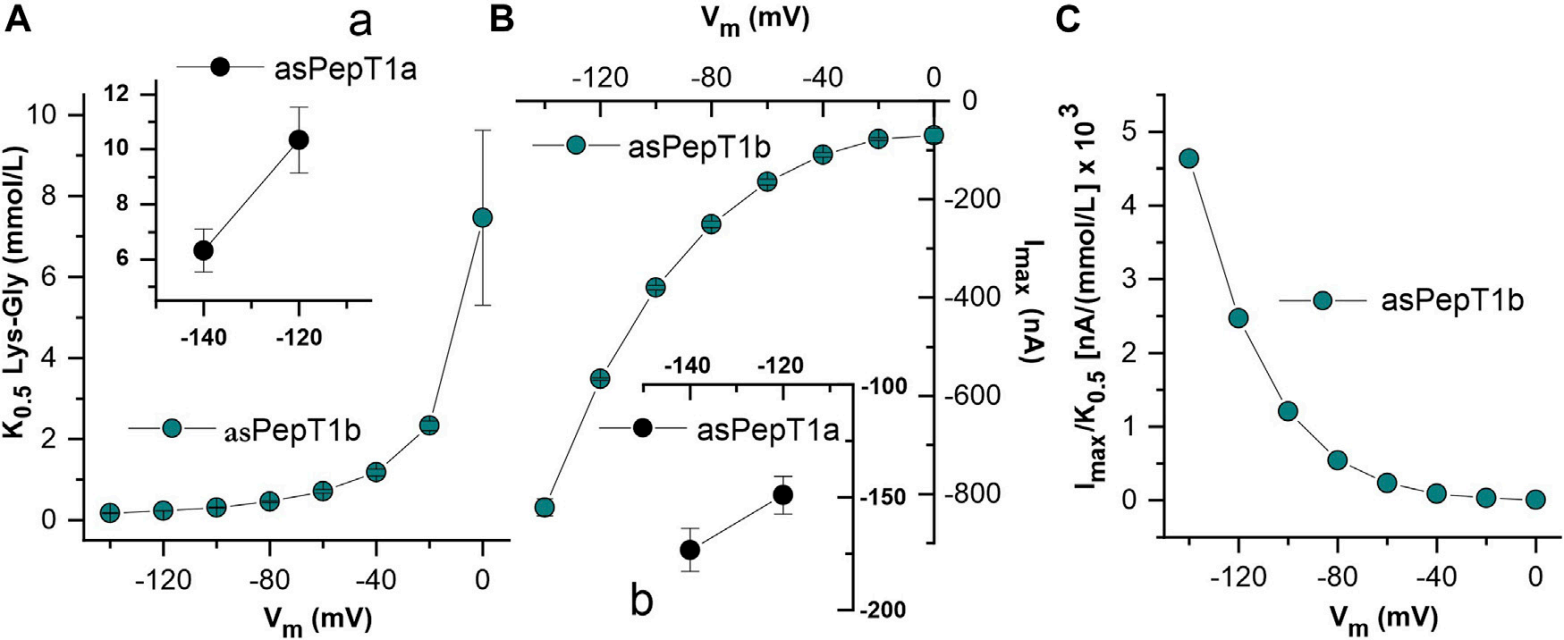
Dose-response analysis of Atlantic salmon PepT1b: *K*_0.5_, *I*_max_ and transport efficiency, respectively in **(A,B,C)** evaluated in the presence of Lys-Gly. The current values (Figure 6) were subsequently fitted with the Michaelis-Menten equation to obtain the apparent relative affinity (1/*K*_0.5_) and the maximal relative current (*I*_max_) and transport efficiency (*I*_max_/*K*_0.5_) at each indicated voltage and at pH 7.6. In **(A,B)**, the inserts **(A)**, **(a)** and **(B)**, **(b)** represent the estimations of kinetic parameters at −120 mV and −140 mV for the Atlantic salmon PepT1a transporter.

Kinetic parameters were determined by fitting current vs. substrate concentration with the Michaelis-Menten equation (Eq. 1). For Atlantic salmon PepT1b, the relative apparent affinity (1/*K*_0.5_) values are reported as a function of potential (Figure 7A). By changing the membrane potential from −140 mV to 0 mV, the *K*_0.5_ values increase progressively from the minimal value of 0.18 ± 0.01 mmol/L at −140 mV to the maximal value of 7.46 ± 2.16 mmol/L recorded at 0 mV. The maximal relative current is influenced by membrane potential with the maximal value reported of −827.24 ± 17.33 nA at −140 mV (Figure 7B). Due to the absence of transport saturation for PepT1a, only a few kinetic parameters were estimated at −140 and −120 mV, as reported in the insert in Figure 7. In Figure 7Aa, the estimated values of relative apparent affinity (10.36 ± 1.19 mmol/L at −120 mV and 6.33 ± 0.78 mmol/L at −140 mV) and in Figure 7Bb the values of the maximal relative current (−149.07 ± 8.26 nA at −120 mV and −173.34 ± 9.54 nA at −140 mV) are reported. The transport efficiency was calculated only for PepT1b and is reported in Figure 7C.

For zebrafish PepT1a, the dose-response experiments were performed in the presence of the cationic dipeptide Lys-Gly and the anionic dipeptide Gly-Asp. To allow comparison, the experimental substrate and pH condition were the same as those reported for PepT1b (Verri et al., 2003). The kinetic parameters for zebrafish PepT1a are summarized in Table 1.

**TABLE 1:** The kinetic parameters of the inwardly-directed transport of cationic and anionic dipeptides via zebrafish PepT1a measured in two-electrode voltage-clamp experiments. All values are expressed as the mean ± SE of *n* oocytes (each oocyte represents an independent observation). *Xenopus laevis* oocytes were voltage-clamped at −60 mV and at −120 mV and perfused with solutions at the indicated pH conditions. The kinetic parameters (*K*_0.5_ and *I*_max_) were calculated by least-square fit to the Michaelis-Menten equation. All amino acids were L-type. The percentage of the zwitterionic form at a given pH was taken from Kottra et al. (2002). To facilitate the comparison, the kinetic parameters of zebrafish PepT1b are reported in the bottom table (*) (Verri et al., 2010).

zfPepT1a			−60 mV		−120 mV		
	pH	% neutral	*I*_max_ nA	*K*_0.5_ mmol/L	*I*_max_ nA	*K*_0.5_ mmol/L	Oocytes (*n*)
Lys-Gly	8.5	67%	−70.51 ± 15.05	11.24 ± 2.88	−135.58 ± 16.15	7.93 ± 1.13	4
	7.6	17%	−80.55 ± 11.09	18.35 ± 3.51	−123.29 ± 13.65	10.78 ± 1.92	4
Gly-Asp	7.6	<1%	−397.70 ± 92.21	5.89 ± 2.14	−695.23 ± 227.53	5.17 ± 2.04	4
	5.5	8%	−173.23 ± 5.47	0.27 ± 0.03	−260.31 ± 26.04	0.66 ± 0.16	4
*zfPepT1b					−60 mV		

	pH	% Neutral	*I*_max_ nA	*K*_0.5_ mmol/L
Lys-Gly	8.5	67%	−252 ± 14	3.5 ± 1.26
	7.6	17%	−440 ± 20	16 ± 1.26
Gly-Asp	7.6	<1%	−128 ± 28	21 ± 5
	5.5	8%	−94 ± 8	0.21 ± 0.03

## 4 Discussion

### 4.1 Dipeptides containing a charged amino acid are transported by PepT1a and PepT1b

Several studies document that not only neutral dipeptides but also dipeptides carrying net positive or negative charges are transported by PepT1 and induce inward transport currents. Although it is still controversial how PepT1 handles differently charged substrates together with H^+^ and the stoichiometry of the transport, it is worth to note that positively and negatively charged dipeptides are all transported by an electrogenic process that follows Michaelis-Menten kinetics. Moreover, electrophysiological studies on mammalian PepT1 and fish PepT1b transporters already reported that the position of the charged amino acid in a peptide significantly affects the transport process (Amasheh et al., 1997; Kottra et al., 2002; Verri et al., 2010; Bossi et al., 2011; Margheritis et al., 2013). Here, by mainly investigating on zebrafish PepT1a and Atlantic salmon PepT1a and PepT1b transporters, the transport current of 1 mmol/L of two negatively charged dipeptides (Asp-Gly and Gly-Asp) and of two pairs of positively charged dipeptides (Lys-Gly and Gly-Lys; Lys-Met and Met-Lys) were recorded and compared (see Figure 2 for reference). Transport currents show differences in the capacity of PepT1a and PepT1b to handle charged dipeptides. In particular, the transport currents of the negatively charged dipeptides Asp-Gly and Gly-Asp relate to the position of the charged amino acid in PepT1a but not in PepT1b transporters. Conversely, the position of the charged residue in the positive substrates defines the current amplitude in Atlantic salmon PepT1b. Notably, this effect is also observed in rabbit PepT1, seabass PepT1b and zebrafish PepT1b, even if in the latter only for the Lys-Gly and Gly-Lys pair (Margheritis et al., 2013).

### 4.2 The charge position in the dipeptide and the amplitude of the transport current

In PepT1a, the currents elicited by the dipeptides with a positive residue are smaller than that of Gly-Gln; in particular, less than 20% in zebrafish for all dipeptides and in Atlantic salmon for Lys-Gly and Gly-Lys. But, in Atlantic salmon PepT1a, the transport currents of Lys-Met and Met-Lys are reduced to ∼50% of the Gly-Gln current. Thus, the position of the positive residue in the dipeptide slightly impacts on the activities of PepT1a transporters. In zebrafish PepT1a, when the negative charge is in the N-terminal position (Asp-Gly), the transport current increases (see Figure 2A for reference) and is considerably influenced by the voltage (see Figure 3A for reference). In Atlantic salmon PepT1a, the amplitude of the current in the presence of Asp-Gly is higher than that in the presence of Gly-Gln, although in a limited voltage range of ∼ −60 mV. At −140 mV a slight decrease of current is recorded with respect to the current due to Gly-Gln. For both PepT1a transporters, when the negative charge is in the C-terminal position (Gly-Asp), the transport currents are like Gly-Gln current at −60 mV (see Figure 2A for reference).

It is worth to note that these functional data on PepT1a proteins are supported by the evidence that a highly conserved Lys (instead of a Gln) residue is present in both Atlantic salmon and zebrafish PepT1a transporters immediately downstream transmembrane domain 7, in a “virtually” extracellular region where some residues involved in the transporter binding pocket have been defined. As previously described for the bacterial *Yersinia enterocolitica* PepT (YePEPT)—where site-directed mutagenesis of Lys^314^ (corresponding to Gln^300^ in human PepT1, Gln^307^ in zebrafish PepT1b and Gln^306^ in Atlantic salmon PepT1b) did tune the substrate specificity of the transporter (Boggavarapu et al., 2015)—the presence of a positive Lys residue [Lys^310^ in zebrafish PepT1a and Lys^306^ in Atlantic salmon PepT1a; for details see, e.g., the alignments reported in Vacca et al. (2019), and in Gomes et al. (2020)] close to the binding pocket is responsible of an electrostatic interaction with the negatively charged substrate that consequently increases the transport of such substrates and reduces the specificity for the positively charged substrate. Thus, the data presented here not only confirm the crucial role in the recognition and specificity of a specific residue, but also highlight the value of employing a comparative approach that utilizes “natural variants” to elucidate the structure-function relationship of a membrane protein. In this respect, a comprehensive structure-function analysis, which is out of the scope of this paper, and a detailed evaluation of the entire set of protein amino acids that do interact with the present set of substrates is required.

In PepT1b, the negatively charged substrates elicit currents that are always smaller than Gly-Gln at all tested voltages, particularly in zebrafish, where the relative currents are very small (see Figures 2A, 3A for reference). To date, it is not easy to explain this behavior. However, it is reported that negatively charged dipeptides are transported in uncharged form, but a small fraction of them can be transiently protonated in the very proximity of the membrane and thus translocated virtually as charged species together with two protons (Kottra et al., 2002). At pH 7.6 less than 1% of Asp-Gly and of Gly-Asp are present in the electrical neutral form (see Supplementary Table S1). Thus, the largest inward current recorded for Asp-Gly but not for Gly-Asp in PepT1a proteins could be ascribed to the easy protonation of the negatively charged form in the proximity of the binding site as a function of the charge position in the dipeptide. The percentage of the negatively charged dipeptide Gly-Asp in the neutral form increases from <0.1 to 8% upon a pH reduction from 7.5 to 5.5.

### 4.3 The effect of the pH on the kinetic parameters of charged dipeptide transport

Based on previously published data in rabbit PepT1 and zebrafish PepT1b, the reduction of pH leads to a marked increase in binding affinity (ratio: ∼39 in rabbit PepT1 and ∼100 in zebrafish PepT1b), whereas the simultaneous changes in *I*_max_ are only moderate in both transporters (ratio: ∼0.7). Notably, by changing the external pH from 7.5 to 5.5 an increase in *I*_max_ occurs in rabbit PepT1 while a decrease in *I*_max_ is observed in zebrafish PepT1b, according to its peculiar pH dependency (Margheritis et al., 2013). In zebrafish PepT1a upon a reduction of pH from 7.6 to 5.5, the apparent affinity increases (ratio: ∼22), whereas a marked decrease in *I*_max_ is reported (ratio: ∼2.3). Data on Gly-Asp kinetic parameters of zebrafish PepT1a suggest that at pH 5.5 the substrate apparent affinity is similar between PepT1a (*K*_0.5_ 0.27 ± 0.03 mmol/L) and PepT1b [*K*_0.5_ 0.21 ± 0.03 mmol/L; (Verri et al., 2010)]. When the external pH is increased at 7.5–7.6, the apparent affinity decreases in both transporters: in PepT1a to a *K*_0.5_ 5.89 ± 2.14 mmol/L, and in PepT1b, more strongly, to a *K*_0.5_ 21 ± 5 mmol/L (Verri et al., 2010).

In this context, it is worth to note that the different pH values here investigated refer to the extracellular conditions naturally occurring in fish guts, where the presence and/or contribution of the Na^+^/H^+^ exchanger in modifying the pH in the proximity of the brush border may be a species-specific function [see, e.g., (Verri et al., 1992; Maffia et al., 1997; Verri et al., 2000; Thwaites et al., 2002; Watanabe et al., 2005; Wang et al., 2017)].

### 4.4 The transport of Lys-containing dipeptides

The results on the transport currents of positively charged substrates reveal that in Atlantic salmon PepT1b the substrates carrying the charged residue in the N-terminal position (Lys-Gly and Lys-Met) elicit significantly higher currents than Gly-Gln and the reversed dipeptides (Gly-Lys and Met-Lys) (see Figures 2B, C for reference). The *I*/*V* relationship for Atlantic salmon PepT1b shows that the amplitude of the Lys-Gly transport-associated current increases when the charged amino acid is in the N-terminal position and diminishes in the presence of Gly-Lys if compared to the Gly-Gln current (see Figure 3B for reference). Although in zebrafish PepT1b the Lys-Gly transport current has larger amplitude than the Gly-Lys transport current, the amplitude generated by both substrates is significantly lower than the reference current (see Figure 2B for reference) at all the tested voltages (see Figure 3B for reference). Data obtained by the transport activity of zebrafish PepT1b show similar currents between Met-Lys, Lys-Met and Gly-Gln (see Figure 3C for reference). For both PepT1a transporters, the currents in the presence of the four positively charged substrates are smaller than the reference current (see Figures 2B, C for reference). Moreover, the *I*/*V* relationships for Lys-Gly and Gly-Lys clearly show that these substrates are almost unable to elicit significant currents in the tested voltage range (see Figure 3B for reference). Notably, in rabbit PepT1, the electrophysiological recordings combined with simultaneous intracellular pH measurement under voltage-clamp conditions show that Lys-Gly can be transported both in charged and neutral form (Kottra et al., 2002), whereas Gly-Lys is transported in neutral form. Although the differences in the current amplitudes between the transport of Lys-Gly and Gly-Lys could be due to differences in substrate affinity, the results from the rabbit transporter explain the excess in transport current observed in Atlantic salmon PepT1b for Lys-Gly. Moreover, the Lys-Gly dose-response experiments highlight the difference in transport kinetics between Atlantic salmon PepT1a and PepT1b.

### 4.5 The effect of voltages and pH on the transport of Lys-containing dipeptides

The fitting with the Michaelis-Menten equation allows the computation of the kinetic parameters (*K*_0.5_ and *I*_max_) at each voltage for PepT1b (see Figures 7A, B for reference), but not for PepT1a. In our hands, the current recorded in PepT1a does not reach the saturation (see Figure 6A for reference), and residual pre-steady state currents are visible even with perfusion of Lys-Gly 30 mmol/L (Figure 6C), making the evaluation of the kinetic parameters not reliable. In the zebrafish transporters, the comparison between *K*_0.5_ values at the same experimental conditions (pH 7.6 and at −60 mV) shows that PepT1b transports Lys-Gly with an affinity five-fold higher (*K*_0.5_ 3.5 ± 0.6 mmol/L; see Verri et al., 2010) than PepT1a (*K*_0.5_ 18.35 ± 3.51) (Table 1). Moreover, when the external pH is increased from 7.5–7.6 to 8.5 the percentage of the positively charged Lys-Gly molecules decreases from 83% to 33% (see Supplementary Table S1) and the kinetic parameters are differently influenced in PepT1b and PepT1a transporters. Zebrafish PepT1b shows a decrease in relative affinity (ratio: ∼0.2) but an increase in maximal relative current (ratio: ∼0.6), whereas PepT1a shows an increase in affinity (ratio: ∼1.6) but the simultaneous change in maximal relative current is only moderate (ratio: ∼1.1).

### 4.6 The role of PepT1a and PepT1b in nutrition

In this paper, the transport data on Lys- and Met-containing substrates have been evaluated also in the perspective of better evaluating the role of piscine PepT1-type transporters in nutrient uptake. Applying a pulse protocol, the transport current of 1 mmol/L of each dipeptide was measured at pH 6.5 and 7.6 and compared to evaluate both voltage and pH dependency (see Figure 4 for reference). The same experimental procedure was applied by Margheritis et al. (2013) to collect the transport current of zebrafish PepT1b, seabass PepT1b, and mammalian transporters (Margheritis et al., 2013). When the results obtained for Atlantic salmon PepT1b are compared to the other transporters, the *I*/*V* relationships of Lys-Gly show similar behavior to those found for zebrafish transporter, whereas the *I*/*V* relationships of Met-Lys and Lys-Met appear to be like those observed for seabass and mammalian transporters (Margheritis et al., 2013). When the current amplitudes are compared between PepT1a and PepT1b transporters of each species, the highest values are obtained with PepT1b. These results suggest the low involvement of PepT1a in the transport of these important nutritional dipeptides. Summarizing for PepT1a transporters, dipeptides containing Lys and/or Met show currents smaller than the Gly-Gln reference current, for all the tested potentials and pH conditions. Conversely, large currents are shown in Atlantic salmon PepT1b for all the tested substrates with the only exception of Gly-Lys. These currents are influenced by membrane potential and by the position of the charged residue in the dipeptide. Particularly in zebrafish PepT1a transporter, the currents elicited by all the tested substrates are slightly increased by membrane potentials and by different pH conditions. In Atlantic salmon PepT1a, the transport current is augmented by increasing negative potential only in the presence of Met-containing dipeptides with a peculiar pH dependency. In Atlantic salmon PepT1b, large currents are observed in the presence of dipeptides carrying Lys in N-terminus position at the more negative potentials and at external pH 7.6. The different transport current amplitudes between the various PepT1-type transporters in the presence of negatively charged substrate can be due to differences in the amino acid residues involved in substrate and/or in H^+^ binding. Regarding the positively charged dipeptides, the current elicited by these substrates in PepT1a are always smaller than in PepT1b.

Until now it is not completely defined if the two transport systems, PepT1a and PepT1b, share (a) physiological role(s) in terms of nutrient absorption and/or molecule sensing, cellular localization in the gut epithelium, as well as the regulation of their expression, but it is well-known that PepT1b proteins are expressed at least one order of magnitude higher than PepT1a proteins in the proximal intestine and are directly involved in dietary protein degradation product (di/tripeptides) uptake (see e.g., Verri et al., 2003; Rønnestad et al., 2010; Vacca et al., 2019; Gomes et al., 2020). Considering that the intestinal epithelium is made of more than one cell type, we do not know whether this difference reflects a difference in the levels of expression of PepT1b vs. PepT1a in the same cell type or it results from the expression of PepT1b in a cell type that is more abundantly represented in the epithelium than (an) other cell type(s). The results here reported show that PepT1a and PepT1b differentially respond to charged dipeptides adding a piece to our knowledge on the functional role of PepT1a transporters in fish, and their involvement in nutrient sensing cannot be excluded. In this context, it is also important to remember that the presence of PepT1a and PepT1b in the intestines of fish is in literature reported to be linked to the variability of their natural environment, their nutritional intake, and the unique characteristics of their digestive systems. Moreover, this variation has been observed in different fish species and can be linked to factors such as food availability, dietary changes, environmental conditions (freshwater or seawater), and gut inflammation (see e.g., Vacca et al., 2019, and literature cited therein). The recent research do suggest that both PepT1a and PepT1b may be expressed and function in teleost fish, responding in similar or different ways to internal and external challenges. Therefore, all these factors should be considered when studying the digestive processes in these organisms. Moreover, we do not know the role of PepT1a and PepT1b in tissues other than the intestine, e.g., PepT1b in zebrafish kidney and spleen and Atlantic salmon brain, and PepT1a in zebrafish ovary (see Verri et al., 2003; Rønnestad et al., 2010; Vacca et al., 2019). And, notably, regarding the functionality and expression in the Atlantic salmon, while PepT1a has been shown to operate in the same post-gastric portions of the intestine where PepT1b also functions (Gomes et al., 2020), its expression in other tissues has been found considerably lower, so that and the functional significance of PepT1a in other tissues remains practically unknown at present and new research is needed to define its potential role(s) in Atlantic salmon physiology.

As a final consideration, the value of transport-associated current collected at membrane potentials lower than–40/-60 mV aim to analyze the electrochemical gradient effect for a deep biophysical characterization of the transport of the different piscine PepT1 proteins here studied.

## 5 Conclusion

Our data on the transport of positively and negatively charged substrates in Atlantic salmon and zebrafish have dual importance. First, the information about the ability of the various (naturally occurring) fish (PepT1a and PepT1b) proteins to transport differently the charged peptides may be crucial in modeling the steps of translocation of charged peptidomimetics after comparison of the functional data to the amino acid sequence and structure of the single proteins. Secondly, PepT1-type transporters are relevant in animal nutrition, being Lys and Met essential amino acids in fish; and considering that Lys- and Met-containing dipeptides are differently treated by the various PepT1a and PepT1b proteins may help in defining a “species-specific” strategy to improve the composition of the feeds used in aquaculture, as for dipeptide-based diets supplementation [see, e.g., (Dabrowski et al., 2010)].

## Data Availability

The original contributions presented in the study are included in the article/Supplementary Material, further inquiries can be directed to the corresponding authors.
