# Supplementary material for: The teleost fish PepT1-type peptide transporters and their relationships with neutral and charged substrates
Source: Front Physiol. 2023 Aug 21;14:1186475. doi: 10.3389/fphys.2023.1186475 (PMC10475540; doi:10.3389/fphys.2023.1186475)
Supplement: Supplementary file 1 [file DataSheet2.PDF]

Statistical Summary Document

Manuscript Title: The teleost fish PepT1-type peptide transporters and their relationships with neutral and charged substrates

Authors: Francesca Vacca1, Ana S. Gomes2*, Marco De Gennaro3, Ivar Rønnestad2*, Tiziano Verri3*, Elena Bossi1*

Animal model used, if applicable: Xenopus laevis oocytes

Underlying hypothesis: This investigation tests the hypothesis that investigated di and tripeptide transporters (asPepT1a; asPepT1b; zfPepT1a and zfPepT1b) elicit different amplitude of current in transport of different charged dipeptides (GD;DG;GK;KG;MK;KM) with respect to the current elicited in the presence of the reference substrate GQ.

Definitions of 'n': [Define 'n'. If definitions differ, please indicate which definition applies to which experimental question number.]

Statistical summary table Figure 2:

Experimental question number	Finding/ conclusion	Experimental variable	Mean value	Units	Standard Deviation	n	Exact P value	Figure/table in which data are presented	Data comparisons	Statistical test	Any other experimental factors	Comments
zfPepT1a		GQ	-1	ratio	0,30071	15					Vh= -60 mV; pH 7.6	
Is GD zfPepT1a inward current magnitude different from GQ zfPepT1a inward current magnitude?	The GD inward currents and the GQ inward current are NOT different	GD	-0,93865	ratio	0,29703	9	6,317500E-01	Fig 2 A	GD vs GQ	Two-sample t test	Vh= -60 mV; pH 7.6	At level 0.05, mean GQ- mean GD is NOT significantly different from 0
Is DG zfPepT1a inward current magnitude different from GQ zfPepT1a inward current magnitude?	The DG inward currents and the GQ inward current are different	DG	-0,42509	ratio	0,11953	10	3,621590E-10	Fig 2 A	DG vs GQ	Two-sample t test	Vh= -60 mV; pH 7.6	At level 0.05, mean GQ- mean DG is significantly different from 0
Is GK zfPepT1a inward current magnitude different from GQ zfPepT1a inward current magnitude?	The GK inward currents and the GQ inward current are different	GK	-0,14641	ratio	0,15791	12	3,171090E-09	Fig 2 B	GK vs GQ	Two-sample t test	Vh= -60 mV; pH 7.6	At level 0.05, mean GQ- mean GK is significantly different from 0
Is KG zfPepT1a inward current magnitude different from GQ zfPepT1a inward current magnitude?	The KG inward currents and the GQ inward current are different	KG	-0,18108	ratio	0,30073	12	1,047990E-04	Fig 2 B	KG vs GQ	Mann Whitney test	Vh= -60 mV; pH 7.6	at level 0.05, the two distributions are significantly different
Is MK zfPepT1a inward current magnitude different from GQ zfPepT1a inward current magnitude?	The MK inward currents and the GQ inward current are different	MK	-0,14145	ratio	0,07724	10	8,680890E-09	Fig 2 C	MK vs GQ	Two-sample t test	Vh= -60 mV; pH 7.6	At level 0.05, mean GQ- mean MG is significantly different from 0
Is KM zfPepT1a inward current magnitude different from GQ zfPepT1a inward current magnitude?	The KM inward currents and the GQ inward current are different	KM	-0,09968	ratio	0,11191	9	2,065080E-09	Fig 2 C	KM vs GQ	Two-sample t test	Vh= -60 mV; pH 7.6	At level 0.05, mean GQ- mean KM is significantly different from 0
zfPepT1b		GQ	-1	ratio	0,15358	29					Vh= -60 mV; pH 7.6	
Is GD zfPepT1b inward current magnitude different from GQ zfPepT1b inward current magnitude?	The GD inward currents and the GQ inward current are different	GD	-0,12982	ratio	0,0441	9	1,533300E-25	Fig 2 A	GD vs GQ	Two-sample t test	Vh= -60 mV; pH 7.6	At level 0.05, mean GQ- mean GD is significantly different from 0
Is DG zfPepT1b inward current magnitude different from GQ zfPepT1b inward current magnitude?	The DG inward currents and the GQ inward current are different	DG	-0,10691	ratio	0,03507	9	6,185680E-26	Fig 2 A	DG vs GQ	Two-sample t test	Vh= -60 mV; pH 7.6	At level 0.05, mean GQ- mean DG is significantly different from 0
Is GK zfPepT1b inward current magnitude different from GQ zfPepT1b inward current magnitude?	The GK inward currents and the GQ inward current are different	GK	-0,3049	ratio	0,17292	9	1,231810E-13	Fig 2 B	GK vs GQ	Two-sample t test	Vh= -60 mV; pH 7.6	At level 0.05, mean GQ- mean GK is significantly different from 0
Is KG zfPepT1b inward current magnitude different from GQ zfPepT1b inward current magnitude?	The KG inward currents and the GQ inward current are different	KG	-0,63181	ratio	0,13057	9	1,565490E-07	Fig 2 B	KG vs GQ	Two-sample t test	Vh= -60 mV; pH 7.6	At level 0.05, mean GQ- mean KG is significantly different from 0
Is MK zfPepT1b inward current magnitude different from GQ zfPepT1b inward current magnitude?	The MK inward currents and the GQ inward current are NOT different	MK	-1,05314	ratio	0,16725	10	3,620800E-01	Fig 2 C	MK vs GQ	Two-sample t test	Vh= -60 mV; pH 7.6	At level 0.05, mean GQ- mean MK is NOT significantly different from 0
Is KM zfPepT1b inward current magnitude different from GQ zfPepT1b inward current magnitude?	The KM inward currents and the GQ inward current are NOT different	KM	-1,04892	ratio	0,32676	7	7,115800E-01	Fig 2 C	KM vs GQ	Two-sample t test	Vh= -60 mV; pH 7.6	At level 0.05, mean GQ- mean KM is NOT significantly different from 0
asPepT1a		GQ	-1	ratio	0,29716	42					Vh= -60 mV; pH 7.6	
Is GD asPepT1a inward current magnitude different from GQ asPepT1a inward current magnitude?	The GD inward currents and the GQ inward current are NOT different	GD	-0,82976	ratio	0,29948	7	1,675200E-01	Fig 2 A	GD vs GQ	Two-sample t test	Vh= -60 mV; pH 7.6	At level 0.05, mean GQ- mean GD is NOT significantly different from 0
Is DG asPepT1a inward current magnitude different from GQ asPepT1a inward current magnitude?	The DG inward currents and the GQ inward current are different	DG	-1,63575	ratio	0,66728	7	4,532000E-02	Fig 2 A	DG vs GQ	Two-sample t test	Vh= -60 mV; pH 7.6	At level 0.05, mean GQ- mean DG is significantly different from 0
Is GK asPepT1a inward current magnitude different from GQ asPepT1a inward current magnitude?	The GK inward currents and the GQ inward current are different	GK	-0,11277	ratio	0,16684	18	2,063660E-09	Fig 2 B	GK vs GQ	Mann Whitney test	Vh= -60 mV; pH 7.6	at level 0.05, the two distributions are significantly different
Is KG asPepT1a inward current magnitude different from GQ asPepT1a inward current magnitude?	The KG inward currents and the GQ inward current are different	KG	-0,1659	ratio	0,11034	19	7,301180E-10	Fig 2 B	KG vs GQ	Mann Whitney test	Vh= -60 mV; pH 7.6	at level 0.05, the two distributions are significantly different
Is MK asPepT1a inward current magnitude different from GQ asPepT1a inward current magnitude?	The MK inward currents and the GQ inward current are different	MK	-0,61057	ratio	0,15437	17	1,921140E-06	Fig 2 C	MK vs GQ	Mann Whitney test	Vh= -60 mV; pH 7.6	at level 0.05, the two distributions are significantly different
Is KM asPepT1a inward current magnitude different from GQ asPepT1a inward current magnitude?	The KM inward currents and the GQ inward current are different	KM	-0,41582	ratio	0,16007	17	2,627430E-13	Fig 2 C	KM vs GQ	Two-sample t test	Vh= -60 mV; pH 7.6	At level 0.05, mean GQ- mean KM is significantly different from 0
asPepT1b		GQ	-1	ratio	0,30816	38					Vh= -60 mV; pH 7.6	
Is GD asPepT1b inward current magnitude different from GQ asPepT1b inward current magnitude?	The GD inward currents and the GQ inward current are different	GD	-0,74705	ratio	0,31578	8	4,133000E-02	Fig 2 A	GD vs GQ	Two-sample t test	Vh= -60 mV; pH 7.6	At level 0.05, mean GQ- mean GD is significantly different from 0
Is DG asPepT1b inward current magnitude different from GQ asPepT1b inward current magnitude?	The DG inward currents and the GQ inward current are different	DG	-0,60334	ratio	0,19888	8	1,160000E-03	Fig 2 A	DG vs GQ	Two-sample t test	Vh= -60 mV; pH 7.6	At level 0.05, mean GQ- mean DG is significantly different from 0
Is GK asPepT1b inward current magnitude different from GQ asPepT1b inward current magnitude?	The GK inward currents and the GQ inward current are different	GK	-0,2456	ratio	0,32402	15	6,839800E-07	Fig 2 B	GK vs GQ	Mann Whitney test	Vh= -60 mV; pH 7.6	at level 0.05, the two distributions are significantly different
Is KG asPepT1b inward current magnitude different from GQ asPepT1b inward current magnitude?	The KG inward currents and the GQ inward current are different	KG	-2,0322	ratio	0,76367	15	1,163390E-04	Fig 2 B	KG vs GQ	Two-sample t test	Vh= -60 mV; pH 7.6	At level 0.05, mean GQ- mean KG is significantly different from 0
Is MK asPepT1b inward current magnitude different from GQ asPepT1b inward current magnitude?	The MK inward currents and the GQ inward current are NOT different	MK	-1,17158	ratio	0,28611	14	7,573000E-02	Fig 2 C	MK vs GQ	Two-sample t test	Vh= -60 mV; pH 7.6	At level 0.05, mean GQ- mean MK is NOT significantly different from 0
Is KM asPepT1b inward current magnitude different from GQ asPepT1b inward current magnitude?	The KM inward currents and the GQ inward current are different	KM	-2,06651	ratio	0,49139	13	2,102880E-06	Fig 2 C	KM vs GQ	Two-sample t test	Vh= -60 mV; pH 7.6	At level 0.05, mean GQ- mean KM is significantly different from 0

Underlying hypothesis: This investigation tests the hypothesis that investigated di and tripeptide transporters (asPepT1a; asPepT1b; zfPepT1a and zfPepT1b) elicit different amplitude of current in transport of different charged dipeptides (GD;DG;GK;KG;MK;KM) depending on the position of charged residue in the dipeptide (N- vs C-terminal position)

Experimental question number	Finding/ conclusion	Experimental variable	Mean value	Units	Standard Deviation	n	Exact P value	Figure/table in which data are presented	Data comparisons	Statistical test	Any other experimental factors	Comments
Is GD zfPepT1a inward current magnitude different from DG zfPepT1a inward current magnitude?	The GD inward currents and the DG inward current are different	GD	-0,93865	ratio	0,29703	9	7,213060E-08	Fig 2 A	GD vs DG	Two-sample t test	Vh= -60 mV; pH 7.6	At level 0.05, mean GD- mean DG is significantly different from 0
		DG	-0,42509	ratio	0,11953	10						
Is GD zfPepT1b inward current magnitude different from DG zfPepT1b inward current magnitude?	The GD inward currents and the DG inward current are NOT different	GD	-0,12982	ratio	0,0441	9	2,402400E-01	Fig 2 A	GD vs DG	Two-sample t test	Vh= -60 mV; pH 7.6	At level 0.05, mean GD- mean DG is NOT significantly different from 0
		DG	-0,10691	ratio	0,03507	9						
Is GD asPepT1a inward current magnitude different from DG asPepT1a inward current magnitude?	The GD inward currents and the DG inward current are different	GD	-0,82976	ratio	0,29948	7	1,295000E-02	Fig 2 A	GD vs DG	Two-sample t test	Vh= -60 mV; pH 7.6	At level 0.05, mean GD- mean DG is significantly different from 0
		DG	-1,63575	ratio	0,66728	7						
Is GD asepT1b inward current magnitude different from DG asPepT1b inward current magnitude?	The GD inward currents and the DG inward current are NOT different	GD	-0,74705	ratio	0,31578	8	2,944500E-01	Fig 2 A	GD vs DG	Two-sample t test	Vh= -60 mV; pH 7.6	At level 0.05, mean GD- mean DG is NOT significantly different from 0
		DG	-0,60334	ratio	0,19888	8						
Is GK zfPepT1a inward current magnitude different from KG zfPepT1a inward current magnitude?	The GK inward currents and the KG inward current are NOT different	GK	-0,14641	ratio	0,15791	12	8,398600E-01	Fig 2 B	GK vs KG	Mann Whitney test	Vh= -60 mV; pH 7.6	at level 0.05, the two distributions are NOT significantly different
		KG	-0,18108	ratio	0,30073	12						
Is GK zfPepT1b inward current magnitude different from KG zfPepT1b inward current magnitude?	The GK inward currents and the KG inward current are different	GK	-0,3049	ratio	0,17292	9	3,443300E-04	Fig 2 B	GK vs KG	Two-sample t test	Vh= -60 mV; pH 7.6	At level 0.05, mean GK- mean KG is significantly different from 0
		KG	-0,63181	ratio	0,13057	9						
Is GK asPepT1a inward current magnitude different from KG asPepT1a inward current magnitude?	The GK inward currents and the KG inward current are NOT different	GK	-0,11277	ratio	0,16684	18	1,325400E-01	Fig 2 B	GK vs KG	Mann Whitney test	Vh= -60 mV; pH 7.6	at level 0.05, the two distributions are NOT significantly different
		KG	-0,1659	ratio	0,11034	19						
Is GK asepT1b inward current magnitude different from KG asPepT1b inward current magnitude?	The GK inward currents and the KG inward current are different	GK	-0,2456	ratio	0,32402	15	2,329000E-05	Fig 2 B	GK vs KG	Mann Whitney test	Vh= -60 mV; pH 7.6	at level 0.05, the two distributions are significantly different
		KG	-2,0322	ratio	0,76367	15						
Is MK zfPepT1a inward current magnitude different from KM zfPepT1a inward current magnitude?	The MK inward currents and the KM inward current are NOT different	MK	-0,14145	ratio	0,07724	10	3,527400E-01	Fig 2 C	MK vs KM	Two-sample t test	Vh= -60 mV; pH 7.6	At level 0.05, mean MK- mean KM is NOT significantly different from 1
		KM	-0,09968	ratio	0,11191	9						
Is MK zfPepT1b inward current magnitude different from KM zfPepT1b inward current magnitude?	The MK inward currents and the KM inward current are NOT different	MK	-1,05314	ratio	0,16725	10	9,724800E-01	Fig 2 C	MK vs KM	Two-sample t test	Vh= -60 mV; pH 7.6	At level 0.05, mean MK- mean KM is NOT significantly different from 0
		KM	-1,04892	ratio	0,32676	7						
Is MK asPepT1a inward current magnitude different from KM asPepT1a inward current magnitude?	The MK inward currents and the KM inward current are different	MK	-0,61057	ratio	0,15437	17	1,360000E-03	Fig 2 C	MK vs KM	Mann Whitney test	Vh= -60 mV; pH 7.6	at level 0.05, the two distributions are significantly different
		KM	-0,41582	ratio	0,16007	17						
Is MK asepT1b inward current magnitude different from KM asPepT1b inward current magnitude?	The MK inward currents and the KM inward current are different	MK	-1,17158	ratio	0,28611	14	4,359550E-06	Fig 2 C	MK vs KM	Two-sample t test	Vh= -60 mV; pH 7.6	At level 0.05, mean MK- mean KM is significantly different from 0
		KM	-2,06651	ratio	0,49139	13						
