# Supplementary material for: The teleost fish PepT1-type peptide transporters and their relationships with neutral and charged substrates
Source: Front Physiol. 2023 Aug 21;14:1186475. doi: 10.3389/fphys.2023.1186475 (PMC10475540; doi:10.3389/fphys.2023.1186475)
Supplement: Supplementary file 2 [file DataSheet4.PDF]

Statistical Summary Document

Manuscript Title: The teleost fish PepT1-type peptide transporters and their relationships with neutral and charged substrates

Authors: Francesca Vacca1, Ana S. Gomes2*, Marco De Gennaro3, Ivar Rønnestad2*, Tiziano Verri3*, Elena Bossi1*

Animal model used, if applicable: Xenopus laevis oocytes

Underlying hypothesis: This investigation tests the hypothesis that investigated di and tripeptide transporters (asPepT1a; asPepT1b and zfPepT1a) are pH-dependent in trasport of Lysine containing peptides. Moreover it tests if in PepT the increasing of the pH from 6.5 to 7.6 has more impact on the amplitude of transport associated current.

Definitions of 'n': [Define 'n'. If definitions differ, please indicate which definition applies to which experimental question number.]

Statistical summary table Figure 4:

Experimental question number	Finding/ conclusion	Experimental variable	Mean value	Units	Standard Deviation	n	Exact P value	Figure/table in which data are presented	Data comparisons	Statistical test	Any other experimental factors	Comments
Is GK asPepT1b inward current magnitude different between pH 6.5 and pH 7.6 at -140 mV?	The inward currents at pH 6.5 and at pH 7.6 are different	pH 6.5	-137,65279	nA	62,07585	11	0,0003373550	Fig 4 A	pH 6.5 vs pH 7.6	Two-sample t test	Vh= -140 mV	At level 0.05, mean 6.5- mean 7.5 is significantly different from 0
		pH 7.6	-40,87723	nA	25,24021	11					Vh= -140 mV	
Is GK asPepT1b inward current magnitude different between pH 6.5 and pH 7.6 at -60 mV?	The inward currents at pH 6.5 and at pH 7.6 are different	pH 6.5	-29,1459	nA	14,89869	11	0,0001392320	Fig 4 A	pH 6.5 vs pH 7.6	Two-sample t test	Vh= -60 mV	At level 0.05, mean 6.5- mean 7.5 is significantly different from 0
		pH 7.6	-2,82471	nA	2,93599	11					Vh= -60 mV	
Is GK asPepT1b inward current magnitude different between pH 6.5 and pH 7.6 at -20 mV?	The inward currents at pH 6.5 and at pH 7.6 are different	pH 6.5	-8,97871	nA	6,22965	11	0,0011200000	Fig 4 A	pH 6.5 vs pH 7.6	Two-sample t test	Vh= -20 mV	At level 0.05, mean 6.5- mean 7.5 is significantly different from 0
		pH 7.6	-0,50546	nA	3,12214	11					Vh= -20 mV	
Is KG asPepT1b inward current magnitude different between pH 6.5 and pH 7.6 at -140 mV?	The inward currents at pH 6.5 and at pH 7.6 are different	pH 6.5	-482,6239	nA	100,90287	11	0,0003155780	Fig 4 B	pH 6.5 vs pH 7.6	Two-sample t test	Vh= -140 mV	At level 0.05, mean 6.5- mean 7.5 is significantly different from 0
		pH 7.6	-705,30883	nA	130,97725	10					Vh= -140 mV	
Is KG asPepT1b inward current magnitude different between pH 6.5 and pH 7.6 at -60 mV?	The inward currents at pH 6.5 and at pH 7.6 are NOT different	pH 6.5	-115,16017	nA	24,07606	11	0,1870800000	Fig 4 B	pH 6.5 vs pH 7.6	Two-sample t test	Vh= -60 mV	At level 0.05, mean 6.5- mean 7.5 is NOT significantly different from 0
		pH 7.6	-101,656	nA	20,79644	10					Vh= -60 mV	
Is KG asPepT1b inward current magnitude different between pH 6.5 and pH 7.6 at -20 mV?	The inward currents at pH 6.5 and at pH 7.6 are different	pH 6.5	-40,01214	nA	7,76126	11	0,0000680714	Fig 4 B	pH 6.5 vs pH 7.6	Two-sample t test	Vh= -20 mV	At level 0.05, mean 6.5- mean 7.5 is significantly different from 0
		pH 7.6	-24,96171	nA	5,52555	10					Vh= -20 mV	
Is MK asPepT1b inward current magnitude different between pH 6.5 and pH 7.6 at -140 mV?	The inward currents at pH 6.5 and at pH 7.6 are different	pH 6.5	-268,58592	nA	69,76586	11	0,0376000000	Fig 4 C	pH 6.5 vs pH 7.6	Two-sample t test	Vh= -140 mV	At level 0.05, mean 6.5- mean 7.5 is significantly different from 0
		pH 7.6	-337,30026	nA	74,86977	11					Vh= -140 mV	
Is MK asPepT1b inward current magnitude different between pH 6.5 and pH 7.6 at -60 mV?	The inward currents at pH 6.5 and at pH 7.6 are different	pH 6.5	-66,19286	nA	18,48727	11	0,0208900000	Fig 4 C	pH 6.5 vs pH 7.6	Two-sample t test	Vh= -60 mV	At level 0.05, mean 6.5- mean 7.5 is significantly different from 0
		pH 7.6	-50,41831	nA	9,67093	11					Vh= -60 mV	
Is MK asPepT1b inward current magnitude different between pH 6.5 and pH 7.6 at -20 mV?	The inward currents at pH 6.5 and at pH 7.6 are different	pH 6.5	-27,65254	nA	7,95952	11	0,0006893700	Fig 4 C	pH 6.5 vs pH 7.6	Two-sample t test	Vh= -20 mV	At level 0.05, mean 6.5- mean 7.5 is significantly different from 0
		pH 7.6	-16,25488	nA	3,12521	11					Vh= -20 mV	
Is KM asPepT1b inward current magnitude different between pH 6.5 and pH 7.6 at -140 mV?	The inward currents at pH 6.5 and at pH 7.6 are different	pH 6.5	-428,82581	nA	105,22229	11	0,0109700000	Fig 4 D	pH 6.5 vs pH 7.6	Two-sample t test	Vh= -140 mV	At level 0.05, mean 6.5- mean 7.5 is significantly different from 0
		pH 7.6	-564,55345	nA	115,49937	10					Vh= -140 mV	

Is KM asPepT1b inward current magnitude different between pH 6.5 and pH 7.6 at -60 mV?	The inward currents at pH 6.5 and at pH 7.6 are NOT different	pH 6.5	-106,61287	nA	32,52097	11					Vh= -60 mV	
		pH 7.6	-95,33992	nA	25,40298	10	0,3905900000	Fig 4 D	pH 6.5 vs pH 7.6	Two-sample t test	Vh= -60 mV	At level 0.05, mean 6.5- mean 7.5 is NOT significantly different from 0
Is KM asPepT1b inward current magnitude different between pH 6.5 and pH 7.6 at -20 mV?	The inward currents at pH 6.5 and at pH 7.6 are different	pH 6.5	-39,80257	nA	11,81142	11					Vh= -20 mV	
		pH 7.6	-27,9248	nA	8,87544	10	0,0182200000	Fig 4 D	pH 6.5 vs pH 7.6	Two-sample t test	Vh= -20 mV	At level 0.05, mean 6.5- mean 7.5 is significantly different from 0
Is GK asPepT1a inward current magnitude different between pH 6.5 and pH 7.6 at -140 mV?	The inward currents at pH 6.5 and at pH 7.6 are NOT different	pH 6.5	-11,86863	nA	17,46665	9					Vh= -140 mV	
		pH 7.6	-1,58028	nA	4,51346	10	0,1203900000	Fig 4 E	pH 6.5 vs pH 7.6	Two-sample t test	Vh= -140 mV	At level 0.05, mean 6.5- mean 7.5 is NOT significantly different from 0
Is GK asPepT1a inward current magnitude different between pH 6.5 and pH 7.6 at -60 mV?	The inward currents at pH 6.5 and at pH 7.6 are NOT different	pH 6.5	-3,4919	nA	4,23307	11					Vh= -60 mV	
		pH 7.6	-1,32541	nA	3,67088	10	0,2276000000	Fig 4 E	pH 6.5 vs pH 7.6	Two-sample t test	Vh= -60 mV	At level 0.05, mean 6.5- mean 7.5 is NOT significantly different from 0
Is GK asPepT1a inward current magnitude different between pH 6.5 and pH 7.6 at -20 mV?	The inward currents at pH 6.5 and at pH 7.6 are NOT different	pH 6.5	-1,66235	nA	2,81567	11					Vh= -20 mV	
		pH 7.6	-0,79108	nA	3,98848	10	0,5669000000	Fig 4 E	pH 6.5 vs pH 7.6	Two-sample t test	Vh= -20 mV	At level 0.05, mean 6.5- mean 7.5 is NOT significantly different from 0
Is KG asPepT1a inward current magnitude different between pH 6.5 and pH 7.6 at -140 mV?	The inward currents at pH 6.5 and at pH 7.6 are different	pH 6.5	-10,36758	nA	8,43194	9					Vh= -140 mV	
		pH 7.6	-29,36759	nA	13,30393	11	0,0014200000	Fig 4 F	pH 6.5 vs pH 7.6	Mann Whitney test	Vh= -140 mV	at level 0.05, the two distributions are significantly different
Is KG asPepT1a inward current magnitude different between pH 6.5 and pH 7.6 at -60 mV?	The inward currents at pH 6.5 and at pH 7.6 are NOT different	pH 6.5	-3,68938	nA	1,19318	11					Vh= -60 mV	
		pH 7.6	-3,37645	nA	1,74003	11	0,4701000000	Fig 4 F	pH 6.5 vs pH 7.6	Mann Whitney test	Vh= -60 mV	at level 0.05, the two distributions are NOT significantly different
Is KG asPepT1a inward current magnitude different between pH 6.5 and pH 7.6 at -20 mV?	The inward currents at pH 6.5 and at pH 7.6 are different	pH 6.5	-2,47662	nA	1,07022	11					Vh= -20 mV	
		pH 7.6	-1,7343	nA	0,62068	11	0,0604400000	Fig 4 F	pH 6.5 vs pH 7.6	Two-sample t test	Vh= -20 mV	At level 0.05, mean 6.5- mean 7.5 is NOT significantly different from 0
Is MK asPepT1a inward current magnitude different between pH 6.5 and pH 7.6 at -140 mV?	The inward currents at pH 6.5 and at pH 7.6 are NOT different	pH 6.5	-87,98953	nA	20,74045	11					Vh= -140 mV	
		pH 7.6	-85,80013	nA	21,62957	11	0,8438300000	Fig 4 G	pH 6.5 vs pH 7.6	Mann Whitney test	Vh= -140 mV	at level 0.05, the two distributions are NOT significantly different
Is MK asPepT1a inward current magnitude different between pH 6.5 and pH 7.6 at -100 mV?	The inward currents at pH 6.5 and at pH 7.6 are different	pH 6.5	-48,59544	nA	9,20941	11					Vh= -100 mV	
		pH 7.6	-36,71227	nA	10,10718	11	0,0086200000	Fig 4 G	pH 6.5 vs pH 7.6	Mann Whitney test	Vh= -100 mV	at level 0.05, the two distributions are significantly different
Is MK asPepT1a inward current magnitude different between pH 6.5 and pH 7.6 at -60 mV?	The inward currents at pH 6.5 and at pH 7.6 are different	pH 6.5	-26,54239	nA	5,59592	11					Vh= -60 mV	
		pH 7.6	-11,92903	nA	3,53696	11	0,0000004458	Fig 4 G	pH 6.5 vs pH 7.6	Mann Whitney test	Vh= -60 mV	at level 0.05, the two distributions are significantly different
Is MK asPepT1a inward current magnitude different between pH 6.5 and pH 7.6 at -20 mV?	The inward currents at pH 6.5 and at pH 7.6 are different	pH 6.5	-12,18428	nA	2,97734	11					Vh= -20 mV	
		pH 7.6	-4,00966	nA	1,06077	11	0,0000013702	Fig 4 G	pH 6.5 vs pH 7.6	Two-sample t test	Vh= -20 mV	At level 0.05, mean 6.5- mean 7.5 is significantly different from 0
Is KM asPepT1a inward current magnitude different between pH 6.5 and pH 7.6 at -140 mV?	The inward currents at pH 6.5 and at pH 7.6 are NOT different	pH 6.5	-62,02975	nA	20,43259	8					Vh= -140 mV	
		pH 7.6	-78,09111	nA	33,73622	11	0,2497700000	Fig 4 H	pH 6.5 vs pH 7.6	Two-sample t test	Vh= -140 mV	At level 0.05, mean 6.5- mean 7.5 is NOT significantly different from 0
Is KM asPepT1a inward current magnitude different between pH 6.5 and pH 7.6 at -60 mV?	The inward currents at pH 6.5 and at pH 7.6 are NOT different	pH 6.5	-15,31044	nA	4,89597	10					Vh= -60 mV	
		pH 7.6	-9,79648	nA	4,92638	11	0,0528100000	Fig 4 H	pH 6.5 vs pH 7.6	Mann Whitney test	Vh= -60 mV	at level 0.05, the two distributions are NOT significantly different
Is KM asPepT1a inward current	The inward currents at pH 6.5	pH 6.5	-6,9587	nA	1,81109	10					Vh= -20 mV	

magnitude different between pH 6.5 and pH 7.6 at -20 mV?	and at pH 7.6 are different	pH 7.6	-3,55635	nA	1,39881	11	0,0001125920	Fig 4 H	pH 6.5 vs pH 7.6	Two-sample t test	Vh= -20 mV	At level 0.05, mean 6.5- mean 7.5 is significantly different from 0
Is GK zfPepT1a inward current magnitude different between pH 6.5 and pH 7.6 at -140 mV?	The inward currents at pH 6.5 and at pH 7.6 are NOT different	pH 6.5	-4,99486	nA	8,72629	9	0,6886600000	Fig 4 I	pH 6.5 vs pH 7.6	Mann Whitney test	Vh= -140 mV	at level 0.05, the two distributions are NOT significantly different
		pH 7.6	-11,94323	nA	15,45814	13					Vh= -140 mV	
Is GK zfPepT1a inward current magnitude different between pH 6.5 and pH 7.6 at -60 mV?	The inward currents at pH 6.5 and at pH 7.6 are NOT different	pH 6.5	-0,80106	nA	1,12321	9	0,2045200000	Fig 4 I	pH 6.5 vs pH 7.6	Mann Whitney test	Vh= -60 mV	at level 0.05, the two distributions are NOT significantly different
		pH 7.6	-3,2711	nA	3,88649	13					Vh= -60 mV	
Is GK zfPepT1a inward current magnitude different between pH 6.5 and pH 7.6 at -20 mV?	The inward currents at pH 6.5 and at pH 7.6 are NOT different	pH 6.5	-0,55989	nA	0,90492	9	0,3165000000	Fig 4 I	pH 6.5 vs pH 7.6	Mann Whitney test	Vh= -20 mV	at level 0.05, the two distributions are NOT significantly different
		pH 7.6	-1,45153	nA	2,70183	13					Vh= -20 mV	
Is KG zfPepT1a inward current magnitude different between pH 6.5 and pH 7.6 at -140 mV?	The inward currents at pH 6.5 and at pH 7.6 are different	pH 6.5	-3,8128	nA	3,69343	8	0,0409000000	Fig 4 L	pH 6.5 vs pH 7.6	Mann Whitney test	Vh= -140 mV	at level 0.05, the two distributions are significantly different
		pH 7.6	-17,62875	nA	20,88912	12					Vh= -140 mV	
Is KG zfPepT1a inward current magnitude different between pH 6.5 and pH 7.6 at -60 mV?	The inward currents at pH 6.5 and at pH 7.6 are NOT different	pH 6.5	-2,70525	nA	1,5875	9	0,5458000000	Fig 4 L	pH 6.5 vs pH 7.6	Mann Whitney test	Vh= -60 mV	At level 0.05, mean 6.5- mean 7.5 is NOT significantly different from 0
		pH 7.6	-2,23669	nA	2,28213	12					Vh= -60 mV	
Is KG zfPepT1a inward current magnitude different between pH 6.5 and pH 7.6 at -20 mV?	The inward currents at pH 6.5 and at pH 7.6 are NOT different	pH 6.5	-1,50626	nA	0,71059	9	0,1351900000	Fig 4 L	pH 6.5 vs pH 7.6	Two-sample t test	Vh= -20 mV	At level 0.05, mean 6.5- mean 7.5 is NOT significantly different from 0
		pH 7.6	-0,24257	nA	2,62399	12					Vh= -20 mV	
Is MK zfPepT1a inward current magnitude different between pH 6.5 and pH 7.6 at -140 mV?	The inward currents at pH 6.5 and at pH 7.6 are NOT different	pH 6.5	-47,14821	nA	20,26927	8	0,3519900000	Fig 4 M	pH 6.5 vs pH 7.6	Two-sample t test	Vh= -140 mV	At level 0.05, mean 6.5- mean 7.5 is NOT significantly different from 0
		pH 7.6	-36,73752	nA	25,38147	11					Vh= -140 mV	
Is MK zfPepT1a inward current magnitude different between pH 6.5 and pH 7.6 at -120 mV?	The inward currents at pH 6.5 and at pH 7.6 are different	pH 6.5	-25,76264	nA	8,84232	10	0,0315700000	Fig 4 M	pH 6.5 vs pH 7.6	Two-sample t test	Vh= -120 mV	At level 0.05, mean 6.5- mean 7.5 is significantly different from 1
		pH 7.6	-16,88222	nA	8,6805	11					Vh= -120 mV	
Is MK zfPepT1a inward current magnitude different between pH 6.5 and pH 7.6 at -60 mV?	The inward currents at pH 6.5 and at pH 7.6 are different	pH 6.5	-12,30504	nA	5,19883	10	0,0002921570	Fig 4 M	pH 6.5 vs pH 7.6	Two-sample t test	Vh= -60 mV	At level 0.05, mean 6.5- mean 7.5 is significantly different from 0
		pH 7.6	-3,28917	nA	1,85441	11					Vh= -60 mV	
Is MK zfPepT1a inward current magnitude different between pH 6.5 and pH 7.6 at -20 mV?	The inward currents at pH 6.5 and at pH 7.6 are different	pH 6.5	-9,46573	nA	2,9687	10	0,0000005580	Fig 4 M	pH 6.5 vs pH 7.6	Two-sample t test	Vh= -20 mV	At level 0.05, mean 6.5- mean 7.5 is significantly different from 0
		pH 7.6	-1,89303	nA	1,6079	11					Vh= -20 mV	
Is KM zfPepT1a inward current magnitude different between pH 6.5 and pH 7.6 at -140 mV?	The inward currents at pH 6.5 and at pH 7.6 are NOT different	pH 6.5	-15,38445	nA	14,21396	11	0,1300300000	Fig 4 N	pH 6.5 vs pH 7.6	Mann Whitney test	Vh= -140 mV	at level 0.05, the two distributions are NOT significantly different
		pH 7.6	-28,2147	nA	19,04923	10					Vh= -140 mV	
Is KM zfPepT1a inward current magnitude different between pH 6.5 and pH 7.6 at -60 mV?	The inward currents at pH 6.5 and at pH 7.6 are NOT different	pH 6.5	-2,86456	nA	1,17752	11	0,1963800000	Fig 4 N	pH 6.5 vs pH 7.6	Two-sample t test	Vh= -60 mV	At level 0.05, mean 6.5- mean 7.5 is NOT significantly different from 0
		pH 7.6	-1,95235	nA	1,89526	10					Vh= -60 mV	
Is KM zfPepT1a inward current magnitude different between pH 6.5 and pH 7.6 at -20 mV?	The inward currents at pH 6.5 and at pH 7.6 are NOT different	pH 6.5	-2,47186	nA	1,32557	9	0,1123500000	Fig 4 N	pH 6.5 vs pH 7.6	Mann Whitney test	Vh= -20 mV	at level 0.05, the two distributions are NOT significantly different
		pH 7.6	-1,75171	nA	1,51201	8					Vh= -20 mV	
