# Supplementary material for: The teleost fish PepT1-type peptide transporters and their relationships with neutral and charged substrates
Source: Front Physiol. 2023 Aug 21;14:1186475. doi: 10.3389/fphys.2023.1186475 (PMC10475540; doi:10.3389/fphys.2023.1186475)
Supplement: Supplementary file 3 [file DataSheet3.PDF]

Statistical Summary Document

Manuscript Title: The teleost fish PepT1-type peptide transporters and their relationships with neutral and charged substrates

Authors: Francesca Vacca1, Ana S. Gomes2*, Marco De Gennaro3, Ivar Rønnestad2*, Tiziano Verri3*, Elena Bossi1*

Animal model used, if applicable: Xenopus laevis oocytes

Underlying hypothesis: This investigation tests the hypothesis that investigated di and tripeptide transporters (asPepT1a; asPepT1b; zfPepT1a and zfPepT1b) are voltage-dependent in trasport of different charged dipeptides (GD;DG;GK;KG)

Definitions of 'n': [Define 'n'. If definitions differ, please indicate which definition applies to which experimental question number.]

Statistical summary table Figure 3:

Experimental question number	Finding/ conclusion	Experimental variable	Mean value	Units	Standard Deviation	n	Exact P value	Figure/table in which data are presented	Data comparisons	Statistical test	Any other experimental factors	Comments
Is GD zfPepT1a inward current magnitude different from GQ zfPepT1a inward current magnitude at -140 mV?	The GD inward currents and the GQ inward current at -140 mV are different	GQ	-1	ratio	0,34474	11	0,0416700000	Fig 3 A	GD vs GQ	Mann Whitney test	Vh= -140 mV	at level 0.05, the two distributions are significantly different
		GD	-0,68706	ratio	0,44681	11					Vh= -140 mV	
Is GD zfPepT1a inward current magnitude different from GQ zfPepT1a inward current magnitude at -40 mV?	The GD inward currents and the GQ inward current at -40 mV are NOT different	GQ	-0,09836	ratio	0,03678	11	0,7927000000	Fig 3 A	GD vs GQ	Mann Whitney test	Vh= -40 mV	at level 0.05, the two distributions are NOT significantly different
		GD	-0,23732	ratio	0,0747	11					Vh= -40 mV	
Is DG zfPepT1a inward current magnitude different from GQ zfPepT1a inward current magnitude at -140 mV?	The DG inward currents and the GQ inward current at -140 mV are different	GQ	-1	ratio	0,34474	11	0,0005091590	Fig 3 A	DG vs GQ	Two-sample t test	Vh= -140 mV	At level 0.05, mean GQ- mean DG is significantly different from 0
		DG	-1,69687	ratio	0,43942	11					Vh= -140 mV	
Is DG zfPepT1a inward current magnitude different from GQ zfPepT1a inward current magnitude at -40 mV?	The DG inward currents and the GQ inward current at -40 mV are different	GQ	-0,09836	ratio	0,03678	11	0,0004972890	Fig 3 A	DG vs GQ	Mann Whitney test	Vh= -40 mV	at level 0.05, the two distributions are significantly different
		DG	-0,09484	ratio	0,05055	11					Vh= -40 mV	
Is GK zfPepT1a inward current magnitude different from GQ zfPepT1a inward current magnitude at -140 mV?	The KG inward currents and the GQ inward current at -140 mV are different	GQ	-1	ratio	0,23333	9	0,0000005953	Fig 3 B	GK vs GQ	Two-sample t test	Vh= -140 mV	At level 0.05, mean GQ- mean GK is significantly different from 0
		GK	-0,01202	ratio	0,05305	7					Vh= -140 mV	
Is GK zfPepT1a inward current magnitude different from GQ zfPepT1a inward current magnitude at -40 mV?	The KG inward currents and the GQ inward current at -40 mV are different	GQ	-0,32705	ratio	0,06624	9	0,0000004042	Fig 3 B	GK vs GQ	Two-sample t test	Vh= -40 mV	At level 0.05, mean GQ- mean GK is significantly different from 0
		GK	-0,00248	ratio	0,00547	9					Vh= -40 mV	
Is KG zfPepT1a inward current magnitude different from GQ zfPepT1a inward current magnitude at -140 mV?	The GK inward currents and the GQ inward current at -140 mV are different	GQ	-1	ratio	0,23333	9	0,0000010129	Fig 3 B	KG vs GQ	Two-sample t test	Vh= -140 mV	At level 0.05, mean GQ- mean KG is significantly different from 0
		KG	-0,02974	ratio	0,04335	9					Vh= -140 mV	
Is KG zfPepT1a inward current magnitude different from GQ zfPepT1a inward current magnitude at -40 mV?	The KG inward currents and the GQ inward current at -40 mV are different	GQ	-0,32705	ratio	0,06624	9	0,0000004980	Fig 3 B	KG vs GQ	Two-sample t test	Vh= -40 mV	At level 0.05, mean GQ- mean KG is significantly different from 0
		KG	-0,01015	ratio	0,0049	9					Vh= -40 mV	
Is GD zfPepT1b inward current magnitude different from GQ zfPepT1b inward current magnitude at -140 mV?	The GD inward currents and the GQ inward current at -140 mV are different	GQ	-1	ratio	0,17513	9	0,0000000738	Fig 3 A	GD vs GQ	Two-sample t test	Vh= -140 mV	At level 0.05, mean GQ- mean GD is significantly different from 0
		GD	-0,11931	ratio	0,05783	9					Vh= -140 mV	
Is GD zfPepT1b inward current magnitude different from GQ zfPepT1b inward current magnitude at -40 mV?	The GD inward currents and the GQ inward current at -40 mV are different	GQ	-0,1205	ratio	0,01776	9	0,0000000191	Fig 3 A	GD vs GQ	Two-sample t test	Vh= -40 mV	At level 0.05, mean GQ- mean GD is significantly different from 1
		GD	-0,01809	ratio	0,00599	9					Vh= -40 mV	
Is DG zfPepT1b inward current magnitude different from GQ	The DG inward currents and	GQ	-1	ratio	0,17513	9					Vh= -140 mV	

zfPepT1b inward current magnitude at -140 mV?	the GQ inward current at -140 mV are different	DG	-0,12834	ratio	0,03578	9	0,0000002080	Fig 3 A	DG vs GQ	Two-sample t test	Vh= -140 mV	At level 0.05, mean GQ- mean DG is significantly different from 0
Is DG zfPepT1b inward current magnitude different from GQ	The DG inward currents and	GQ	-0,1205	ratio	0,01776	9					Vh= -40 mV	
zfPepT1b inward current magnitude at -40 mV?	the GQ inward current at -40 mV are different	DG	-0,01373	ratio	0,00592	9	0,0000000134	Fig 3 A	DG vs GQ	Two-sample t test	Vh= -40 mV	At level 0.05, mean GQ- mean DG is significantly different from 1
Is GK zfPepT1b inward current magnitude different from GQ	The KG inward currents and	GQ	-0,86716	ratio	0,0157	14					Vh= -120 mV	
zfPepT1b inward current magnitude at -120 mV?	the GQ inward current at -120 mV are different	GK	-0,41394	ratio	0,03372	6	0,0006196550	Fig 3 B	GK vs GQ	Mann Whitney test	Vh= -120 mV	At level 0.05, mean GQ- mean GK is significantly different from 0
Is GK zfPepT1b inward current magnitude different from GQ	The KG inward currents and	GQ	-0,27947	ratio	0,01722	14					Vh= -40 mV	
zfPepT1b inward current magnitude at -40 mV?	the GQ inward current at -40 mV are different	GK	-0,10397	ratio	0,00933	6	0,0000000000	Fig 3 B	GK vs GQ	Two-sample t test	Vh= -40 mV	At level 0.05, mean GQ- mean GK is significantly different from 0
Is KG zfPepT1b inward current magnitude different from GQ	The GK inward currents and	GQ	-0,86716	ratio	0,0157	14					Vh= -120 mV	
zfPepT1b inward current magnitude at -120 mV?	the GQ inward current at -120 mV are different	KG	-0,59335	ratio	0,08765	6	0,0006196550	Fig 3 B	KG vs GQ	Mann Whitney test	Vh= -120 mV	At level 0.05, mean GQ- mean KG is significantly different from 0
Is KG zfPepT1b inward current magnitude different from GQ	The KG inward currents and	GQ	-0,27947	ratio	0,01722	14					Vh= -40 mV	
zfPepT1b inward current magnitude at -40 mV?	the GQ inward current at -40 mV are different	KG	-0,13096	ratio	0,01596	6	0,0000000000	Fig 3 B	KG vs GQ	Two-sample t test	Vh= -40 mV	At level 0.05, mean GQ- mean KG is significantly different from 0
Is GD asPepT1a inward current magnitude different from GQ	The GD inward currents and	GQ	-1	ratio	0,53476	7					Vh= -140 mV	
asPepT1a inward current magnitude at -140 mV?	the GQ inward current at -140 mV are different	GD	-0,42522	ratio	0,21705	7	0,0301900000	Fig 3 A	GD vs GQ	Two-sample t test	Vh= -140 mV	At level 0.05, mean GQ- mean GD is significantly different from 0
Is GD asPepT1a inward current magnitude different from GQ	The GD inward currents and	GQ	-0,07076	ratio	0,04612	7					Vh= -40 mV	
asPepT1a inward current magnitude at -40 mV?	the GQ inward current at -40 mV are NOT different	GD	-0,06706	ratio	0,03442	7	0,8675600000	Fig 3 A	GD vs GQ	Two-sample t test	Vh= -40 mV	At level 0.05, mean GQ- mean GD is NOT significantly different from 1
Is DG asPepT1a inward current magnitude different from GQ	The DG inward currents and	GQ	-1	ratio	0,53476	7					Vh= -140 mV	
asPepT1a inward current magnitude at -140 mV?	the GQ inward current at -140 mV are NOT different	DG	-0,8253	ratio	0,37463	7	0,4925300000	Fig 3 A	DG vs GQ	Two-sample t test	Vh= -140 mV	At level 0.05, mean GQ- mean DG is NOT significantly different from 0
Is DG asPepT1a inward current magnitude different from GQ	The DG inward currents and	GQ	-0,07076	ratio	0,04612	7					Vh= -40 mV	
asPepT1a inward current magnitude at -40 mV?	the GQ inward current at -40 mV are NOT different	DG	-0,12584	ratio	0,05881	7	0,0749500000	Fig 3 A	DG vs GQ	Two-sample t test	Vh= -40 mV	At level 0.05, mean GQ- mean DG is NOT significantly different from 1
Is GK asPepT1a inward current magnitude different from GQ	The KG inward currents and	GQ	-1	ratio	0,29188	11					Vh= -140 mV	
asPepT1a inward current magnitude at -140 mV?	the GQ inward current at -140 mV are different	GK	-0,07193	ratio	0,10585	9	0,0000002229	Fig 3 B	GK vs GQ	Two-sample t test	Vh= -140 mV	At level 0.05, mean GQ- mean GK is significantly different from 0
Is GK asPepT1a inward current magnitude different from GQ	The KG inward currents and	GQ	-0,19132	ratio	0,06138	11					Vh= -40 mV	
asPepT1a inward current magnitude at -40 mV?	the GQ inward current at -40 mV are different	GK	-0,01466	ratio	0,02002	11	0,0000009467	Fig 3 B	GK vs GQ	Two-sample t test	Vh= -40 mV	At level 0.05, mean GQ- mean GK is significantly different from 0
Is KG asPepT1a inward current magnitude different from GQ	The GK inward currents and	GQ	-1	ratio	0,29188	11					Vh= -140 mV	
asPepT1a inward current magnitude at -140 mV?	the GQ inward current at -140 mV are different	KG	-0,06283	ratio	0,0511	9	0,0000005785	Fig 3 B	KG vs GQ	Two-sample t test	Vh= -140 mV	At level 0.05, mean GQ- mean KG is significantly different from 0
Is KG asPepT1a inward current magnitude different from GQ	The KG inward currents and	GQ	-0,19132	ratio	0,06138	11					Vh= -40 mV	
asPepT1a inward current magnitude at -40 mV?	the GQ inward current at -40 mV are different	KG	-0,0173	ratio	0,00672	11	0,0000024699	Fig 3 B	KG vs GQ	Two-sample t test	Vh= -40 mV	At level 0.05, mean GQ- mean KG is significantly different from 0
Is GD asPepTb inward current magnitude different from GQ	The GD inward currents and	GQ	-1	ratio	0,42031	8					Vh= -140 mV	
asPepT1b inward current magnitude at -140 mV?	the GQ inward current at -140 mV are NOT different	GD	-0,73523	ratio	0,29322	8	0,1660200000	Fig 3 A	GD vs GQ	Two-sample t test	Vh= -140 mV	At level 0.05, mean GQ- mean GD is NOT significantly different from 0
Is GD asPepT1b inward current magnitude different from GQ	The GD inward currents and	GQ	-0,08811	ratio	0,03351	8					Vh= -40 mV	At level 0.05, mean GQ- mean GD

asPepT1b inward current magnitude at -40 mV?	the GQ inward current at -40 mV are NOT different	GD	-0,06654	ratio	0,02835	8	0,1862000000	Fig 3 A	GD vs GQ	Two-sample t test	Vh= -40 mV	is NOT significantly different from 1
Is DG asPepT1b inward current magnitude different from GQ asPepT1b inward current magnitude at -140 mV?	The DG inward currents and the GQ inward current at -140 mV are different	GQ	-1	ratio	0,42031	8	0,0266200000	Fig 3 A	DG vs GQ	Two-sample t test	Vh= -140 mV	At level 0.05, mean GQ- mean DG is significantly different from 0
		DG	-0,58521	ratio	0,21831	8					Vh= -140 mV	
Is DG asPepT1b inward current magnitude different from GQ asPepT1b inward current magnitude at -40 mV?	The DG inward currents and the GQ inward current at -40 mV are different	GQ	-0,08811	ratio	0,03351	8	0,0186500000	Fig 3 A	DG vs GQ	Two-sample t test	Vh= -40 mV	At level 0.05, mean GQ- mean DG is significantly different from 1
		DG	-0,05327	ratio	0,01578	8					Vh= -40 mV	
Is GK asPepT1b inward current magnitude different from GQ asPepT1b inward current magnitude at -140 mV?	The KG inward currents and the GQ inward current at -140 mV are different	GQ	-1	ratio	0,24683	11	0,0011000000	Fig 3 B	GK vs GQ	Two-sample t test	Vh= -140 mV	At level 0.05, mean GQ- mean GK is significantly different from 0
		GK	-0,58512	ratio	0,26386	11					Vh= -140 mV	
Is GK asPepT1b inward current magnitude different from GQ asPepT1b inward current magnitude at -40 mV?	The KG inward currents and the GQ inward current at -40 mV are different	GQ	-0,17836	ratio	0,05379	11	0,0000343468	Fig 3 B	GK vs GQ	Two-sample t test	Vh= -40 mV	At level 0.05, mean GQ- mean GK is significantly different from 0
		GK	-0,07029	ratio	0,04091	11					Vh= -40 mV	
Is KG asPepT1b inward current magnitude different from GQ asPepT1b inward current magnitude at -140 mV?	The GK inward currents and the GQ inward current at -140 mV are different	GQ	-1	ratio	0,24683	11	0,0000007801	Fig 3 B	KG vs GQ	Two-sample t test	Vh= -140 mV	At level 0.05, mean GQ- mean KG is significantly different from 0
		KG	-2,05148	ratio	0,4289	11					Vh= -140 mV	
Is KG asPepT1b inward current magnitude different from GQ asPepT1b inward current magnitude at -40 mV?	The KG inward currents and the GQ inward current at -40 mV are different	GQ	-0,17836	ratio	0,05379	11	0,0000793376	Fig 3 B	KG vs GQ	Two-sample t test	Vh= -40 mV	At level 0.05, mean GQ- mean KG is significantly different from 0
		KG	-0,29929	ratio	0,06086	11					Vh= -40 mV	
