# Supplementary material for: The teleost fish PepT1-type peptide transporters and their relationships with neutral and charged substrates
Source: Front Physiol. 2023 Aug 21;14:1186475. doi: 10.3389/fphys.2023.1186475 (PMC10475540; doi:10.3389/fphys.2023.1186475)
Supplement: Supplementary file 4 [file DataSheet1.pdf]

The teleost fish PepT1-type peptide transporters and their relationships with neutral and charged substrates

Francesca Vacca, Ana S. Gomes, Marco De Gennaro, Ivar Rønnestad, Elena Bossi, Tiziano Verri

SUPPLEMENTAL MATERIALS

Supplemental Methods

Supplemental Results

Supplemental Figure 1

Supplemental Table 1

(Statistical Summary Documents)

Supplemental Table 2

Supplemental Table 3

Supplemental Table 4

1. Supplemental Methods

1.1 Software and tools

For the present work, the following software/tools were used: AutoDock Vina (Trott & Olson, 2010; Eberhardt et al., 2021), SWISS-MODEL (Bienert et al., 2016), UCSF ChimeraX (Pettersen et al., 2004), PDBfixer (Eastman et al., 2017), OpenBabel (O'Boyle et al., 2011), MGLTools 1.5.6 (Morris et al., 2009), PyMOL (Schrödinger & DeLano, 2020), ChemAxon (https://chemaxon.com/) and BIOVIA Discovery Studio Visualizer (BIOVIA, Dassault Systèmes, Discovery Studio, v. 21.1.0.20298, San Diego: Dassault Systèmes, 2021).

1.2 Protein preparation for docking analysis

The structures of the *Homo sapiens* (human) transporter PepT1 (HsPepT1) selected for this study were obtained from Protein Data Bank (Berman et al., 2003). The PDB files, identified by their PDB codes: 7PN1, 7PMX and 7PMW, represented the apoprotein (Apo) HsPepT1 in the outward facing open conformation, HsPepT1 bound to Ala-Phe in the outward facing open conformation, and the HsPepT1 bound to Ala-Phe in the outward facing occluded conformation, respectively. These structures represent the conformation of the human transporter in three subsequent moments of its transport cycle (Killer et al., 2021).

To prepare structures for docking simulations, the three Protein Data Bank files were processed through PDB fixer (online server), which is able to: i) add missing heavy atoms; ii) add missing hydrogen atoms; iii) build missing loops; iv) convert non-standard residues to their standard equivalents; v) select a single position for atoms with multiple alternate positions listed; vi) delete unwanted chains from the model; vii) delete unwanted heterogens. Afterwards, the prepared structures were uploaded in the AutoDockTools of MGLTools, a phyton-based graphical interface, useful to the preparation of the PDB format files for following Autodock Vina docking analysis. Lastly, by means of the Autodock plugin (Seeliger and de Groot, 2010) available in PyMOL, it was possible to build the docking simulation box for each (i.e., 7PN1, 7PMX and 7PMW) protein model.

1.3 Ligands preparations

For this bioinformatic analysis, the following dipeptides, namely L-Ala-L-Phe (Ala-Phe), Gly-L-Gln (Gly-Gln), L-Asp-Gly (Asp-Gly), Gly-L-Asp (Gly-Asp), Gly-Lys (Gly-Lys), L-Lys-Gly (Lys-Gly), L-Met-L-Lys (Met-Lys) and L-Lys-L-Met (Lys-Met), were downloaded (sdf format) from the PubChem database (Kim et al., 2023). These eight molecules were identified by the following access numbers: Ala-Phe, 96814; Gly-Gln, 123913; Asp-Gly, 151148; Gly-Asp, 97363; Gly-Lys, 3080576; Lys-Gly, 7022320; Met-Lys, 7016112; Lys-Met, 7016114.

We used the ‘Protonation’ feature online tool available in ChemAxon to calculate pK_a and the protonation state of each dipeptide molecule at pH 7.5. On the basis of the calculated values, each dipeptide was modified (by using the BIOVIA Discovery Studio Visualizer) in order to have the model of the most represented charged form at pH 7.5 (for details, see **Supplemental Table 1**). These forms were used for the following molecular docking simulations.

1.4 Protein-ligand complexes and molecular docking simulation

The evaluation of interactions between the HsPepT1 transporter and the selected ligands (Ala-Phe, Gly-Gln, Gly-Asp, Asp-Gly, Gly-Lys, Lys-Gly, Met-Lys, Lys-Met) via molecular docking analysis was carried out by using AutoDockVina. To start the simulations, AutoDock Vina requires that both protein targets and ligands are converted into a digital file format called “pdbqt” (Rizvi et al., 2013), which is a modified protein data bank format containing atomic charges, atom type definitions and, for ligands, topological information (rotatable bonds). Specifically, we run simultaneous multiple ligands docking for each protein by using a PERL script. In particular, we created a .conf file for Vina with an Exhaustiveness of 64 repetitions and an energy maximum range of 4 kcal/mol between the first, lower energy, pose to the tenth, higher energy, pose. All the results were collected for final evaluation of the results.

1.5 Evaluation of the results

After docking analysis, we used the software ChimeraX to generate the ligand-protein complexes of all the best ten docking score interaction for each ligand. For each complex, we generated a graphic representation of the molecular surface of the ligand in the binding pocket of the proteins and the whole ligands in a single complex. All these images are summarized in **Supplemental Figure 1** (for details, see **Supplemental Figure 1A**). To visualize and compare the different binding energies of the ligands to the different conformations of the proteins, all docking binding affinity scores, expressed in Kcal/mol, were recorded into GraphPad Prism software, version 4.02 (GraphPad Software Inc., San Diego, CA, USA). Data are summarized in **Supplemental Figure 1** (for details see, **Supplemental Figure 1B**).

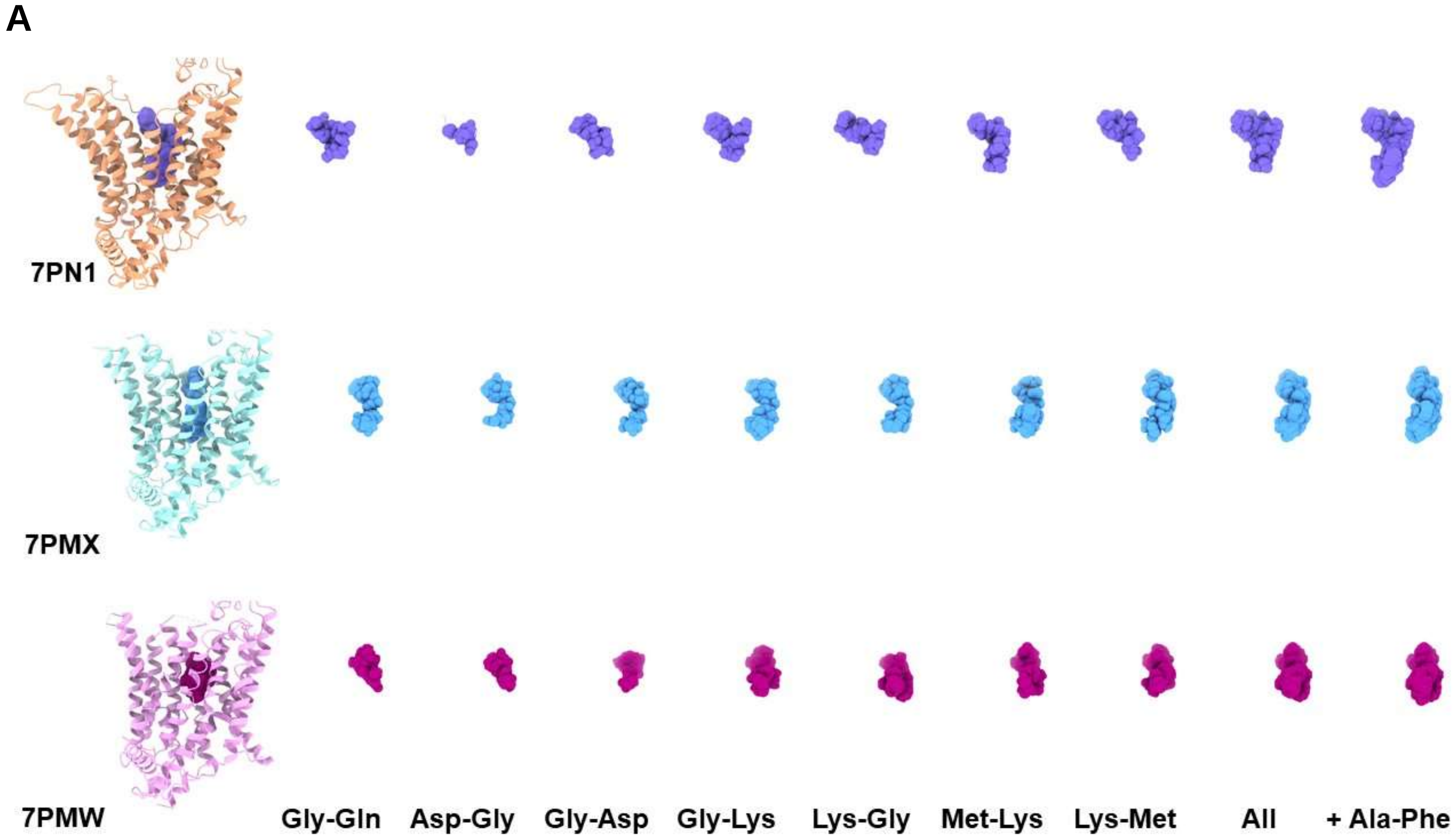
A
7PN1
7PMX
7PMW
Gly-Gln
Asp-Gly
Gly-Asp
Gly-Lys
Lys-Gly
Met-Lys
Lys-Met
All
+ Ala-Phe

B

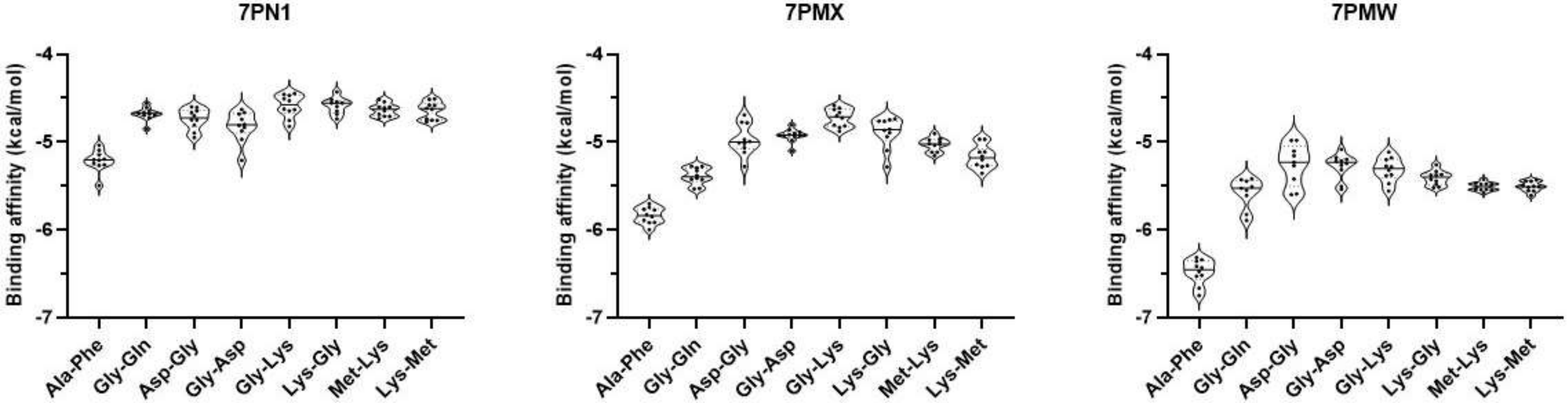

Supplemental Figure 1. (A) ChimeraX analysis of protein-ligand complexes as obtained by molecular docking simulation of Ala-Phe, Gly-Gln, Asp-Gly, Gly-Asp, Gly-Lys, Lys-Gly, Met-Lys and Lys-Met on the human PepT1 transporter in three structural conformations [i.e., the apoprotein (Apo) in the outward facing open conformation, 7PN1, the protein bound to the peptide in the outward facing open conformation, 7PMX, and the protein bound to the peptide in the outward facing occluded conformation, 7PMW]. The figure shows the different positions of the ligands after the calculation of the lower binding energy states in the molecular

docking simulation. **(B)** AutoDock Vina results of the binding affinity (expressed in Kcal/mol) for each predicted ligand-protein complex. Lower energy values indicate a better stability of the ligand-protein complex.

Supplemental Table 1. Percentage of positively, negatively and/or zwitterionic microspecies present at pH 6.5 and 7.6 for each tested dipeptide (see below for details).

		**pH 6.5**					**pH 7.6**				
**3-letter code**	**1-letter code**	**+2**	**+1**	**0**	**-1**	**-2**	**+2**	**+1**	**0**	**-1**	**-2**
**Gly-Gln**	**GQ**	-	-	97.6%	2.3%	-	-	-	77.6%	22.4%	-
**Met-Lys**	**MK**	0.2%	98.5%	1.2%	-	-	-	86.5%	13.2% + 0.2%	-	-
**Lys-Met**	**KM**	0.2%	98.6%	1.2%	-	-	-	87.1%	12.6% + 0.2%	-	-
**Gly-Lys**	**GK**	0.2%	97.5%	2.3%	-	-	-	77.4%	22.3% + 0.2%	-	-
**Lys-Gly**	**KG**	0.2%	98.7%	1.2%	-	-	-	87.2%	12.6% + 0.2%	-	-
**Gly-Asp**	**GD**	-	-	3.6%	94.0%	2.2%	-	-	0.2%	77.4%	22.3%
**Asp-Gly**	**DG**	-	-	-	98.9%	1.0%	-	-	-	89.4%	10.6%

	pH	+2	+1	0	-1	-2
**Gly-Gln**	**6.5**	**-**	**-**	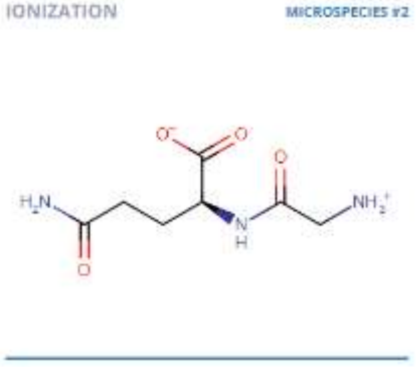<br>**97.6%**	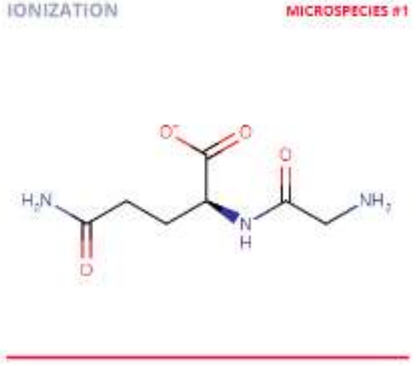<br>**2.3%**	**-**
	**7.6**	**-**	**-**	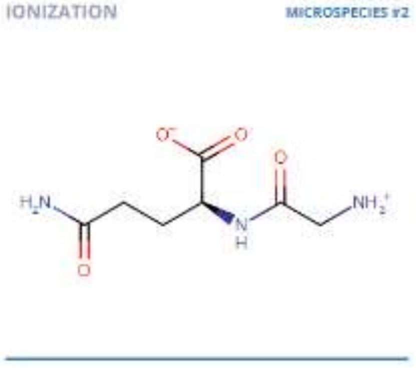<br>**77.6%**	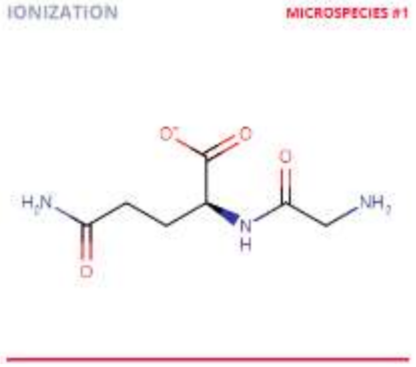<br>**22.4%**	**-**

	pH	+2	+1	0	-1	-2
Met-Lys	6.5	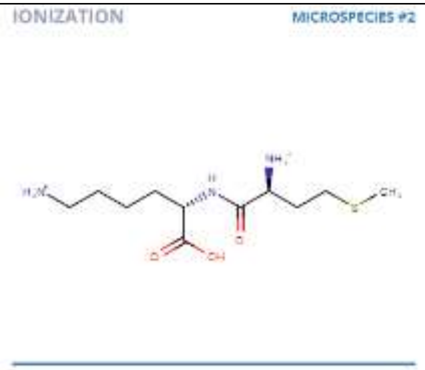 **0.2%**	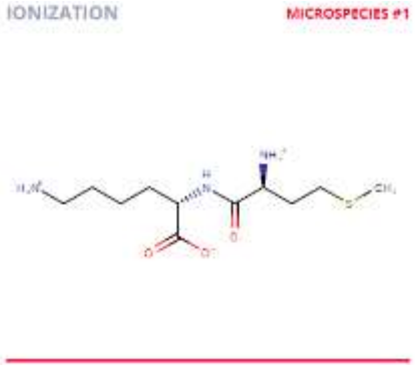 **98.5%**	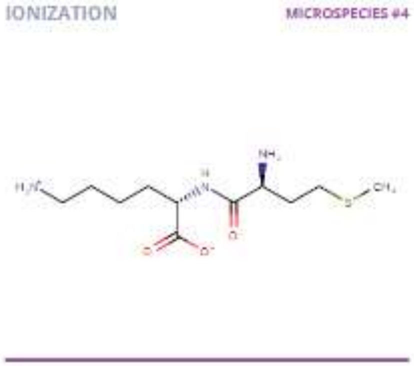 **1.2%**	-	-
	7.6	-	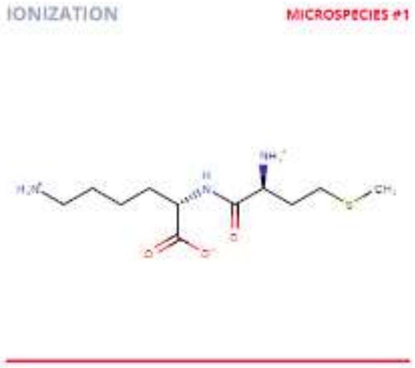 **86.5%**	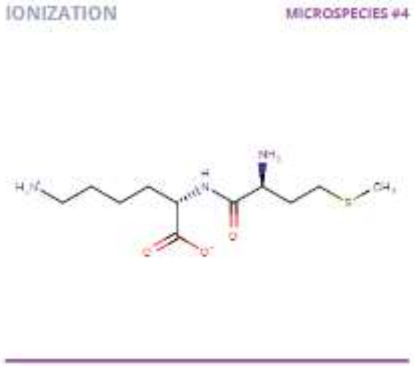 **13.2%** 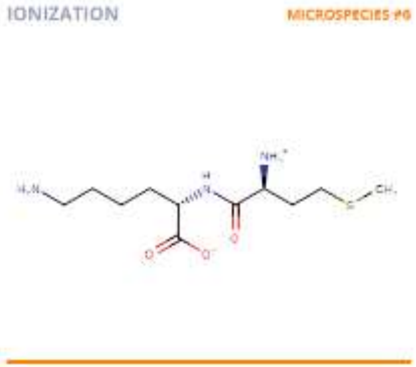 **0.2%**	-	-

<table>
<tr><th></th><th>pH</th><th>+2</th><th>+1</th><th>0</th><th>-1</th><th>-2</th></tr>
<tr><td rowspan="2">Lys-Met</td><td>6.5</td><td>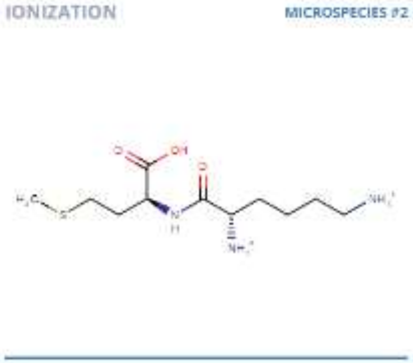

0.2%</td><td>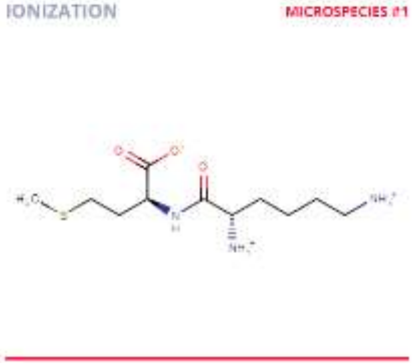

98.6%</td><td>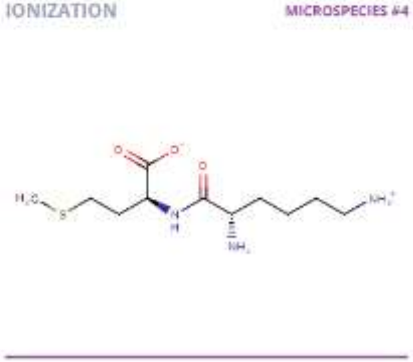

1.2%</td><td>-</td><td>-</td></tr>
<tr><td>7.6</td><td>-</td><td>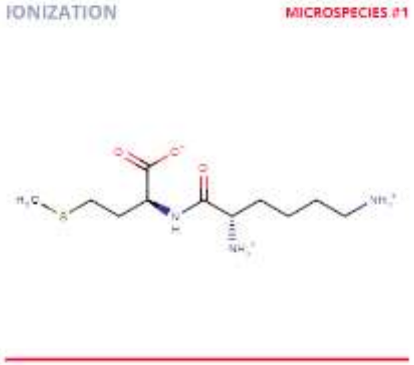

87.1%</td><td>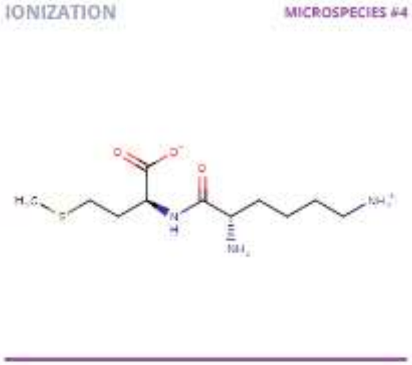

12.6%
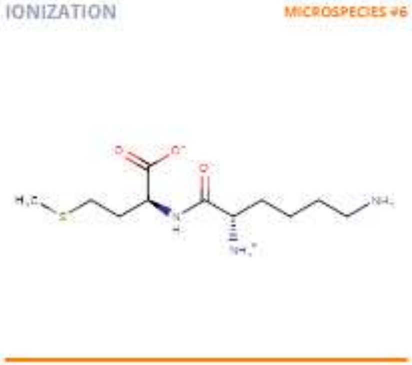

0.2%</td><td>-</td><td>-</td></tr>
</table>

	pH	+2	+1	0	-1	-2
Gly-Lys	6.5	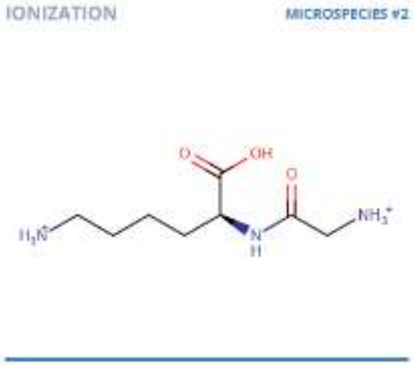 0.2%0	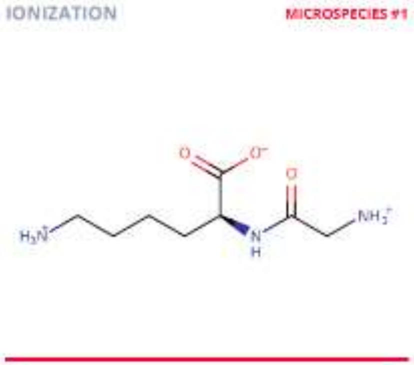 97.5%	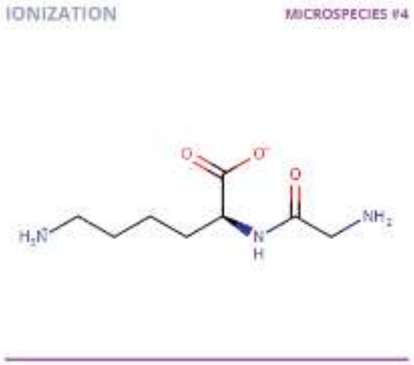 2.3%	-	-
	7.6	-	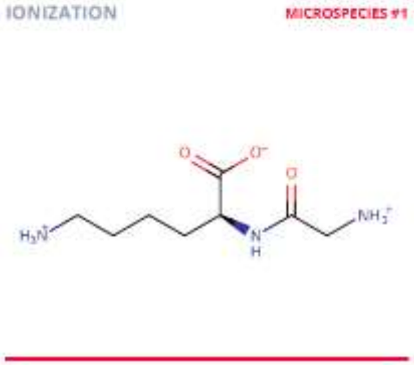 77.4%	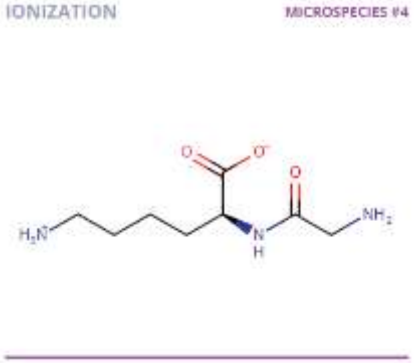 22.6% 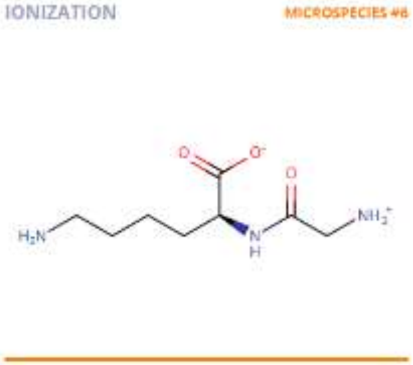 0.2%	-	-

	pH	+2	+1	0	-1	-2
Lys-Gly	6.5	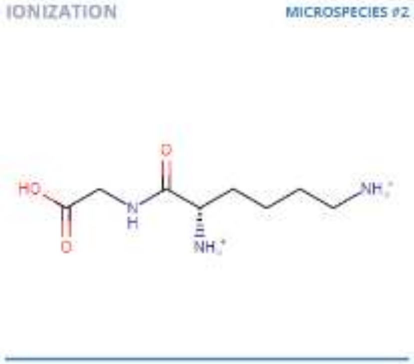<br>0.2%0	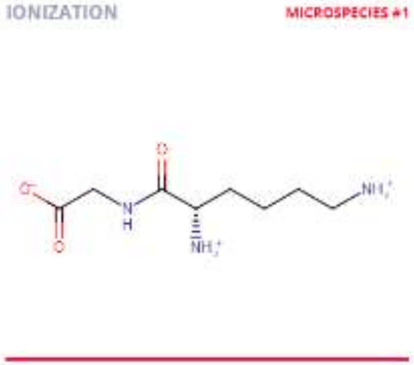<br>98.7%	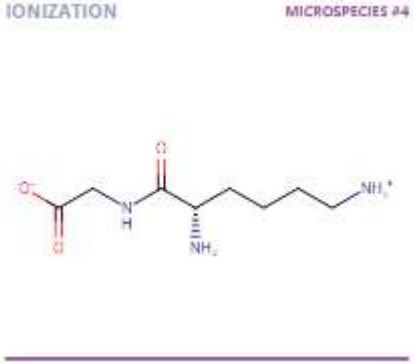<br>1.2%	-	-
	7.6	-	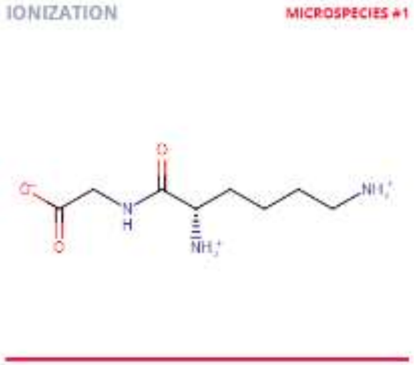<br>87.2%	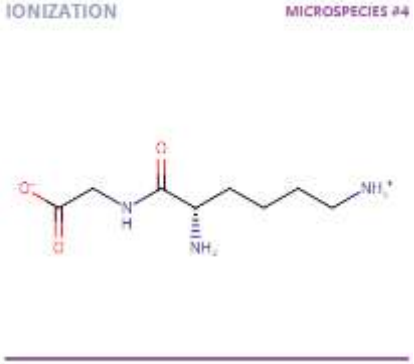<br>12.6%<br>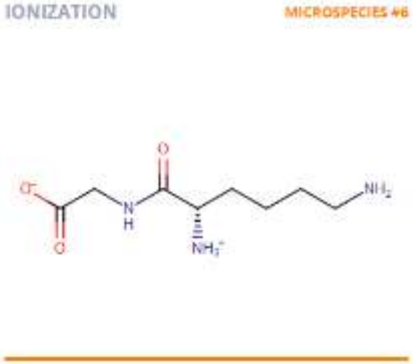<br>0.2%	-	-

	pH	+2	+1	0	-1	-2
**Lys-Gly**	**8.5**	**-**	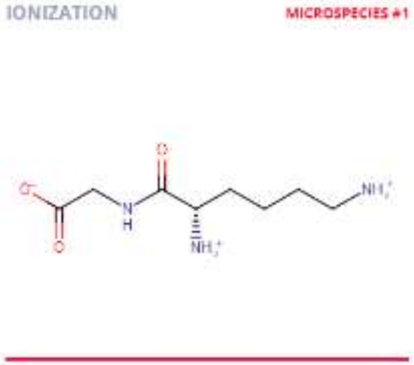 **45.7%**	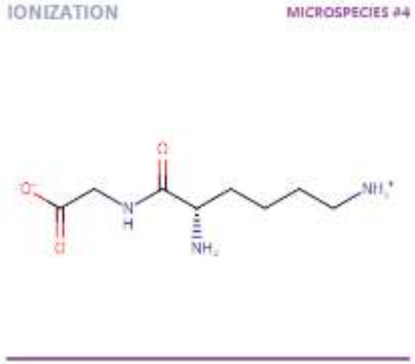 **52.3%** 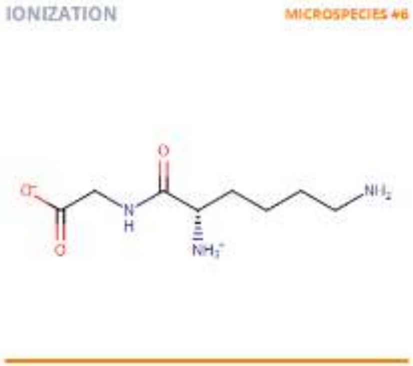 **0.9%**	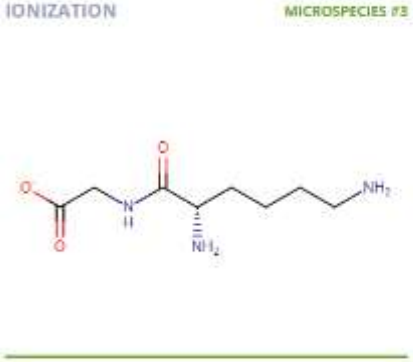 **1.1%**	**-**

	pH	+2	+1	0	-1	-2
**Gly-Asp**	5.5	-	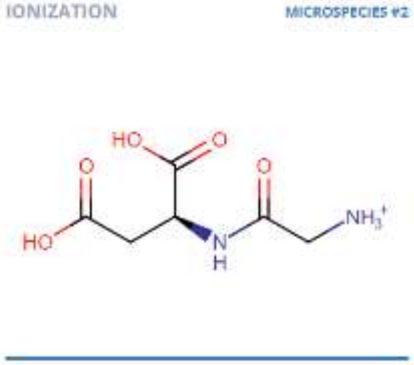<br><br>**0.1%**	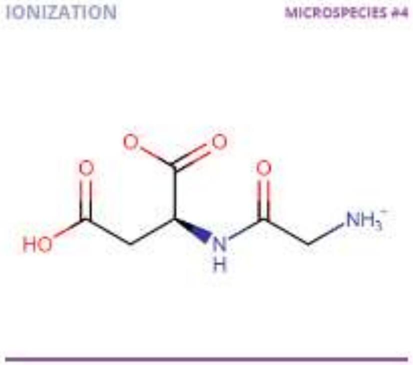<br><br>**27.1%**<br>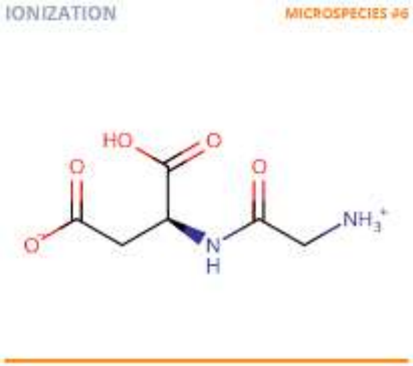<br><br>**0.5%**	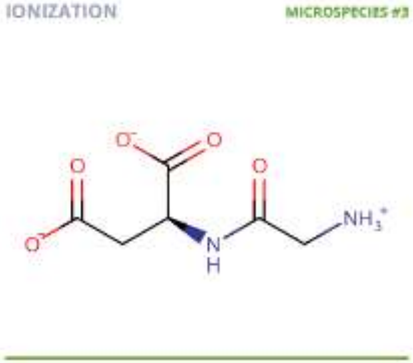<br><br>**72.0%**	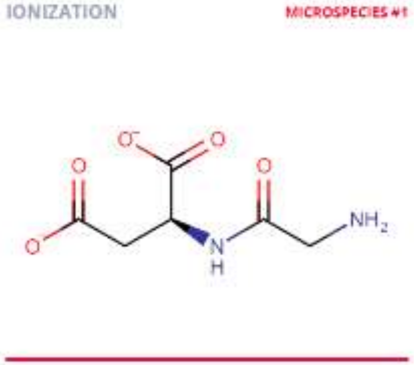<br><br>**0.2%**

	pH	+2	+1	0	-1	-2
Gly-Asp	6.5	-	-	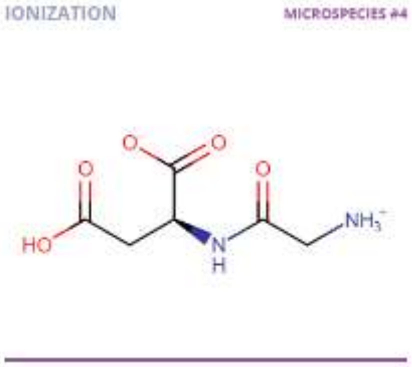<br>3.6%	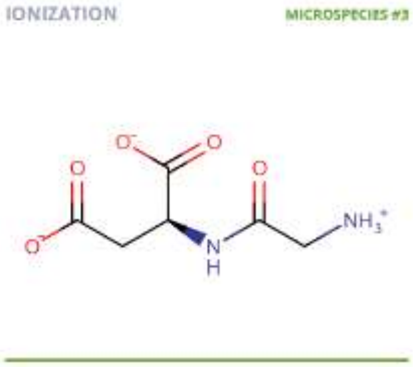<br>94.0%	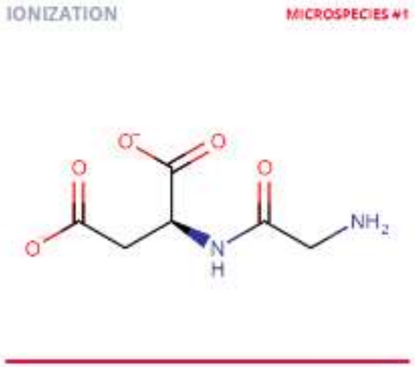<br>2.2%
	7.6	-	-	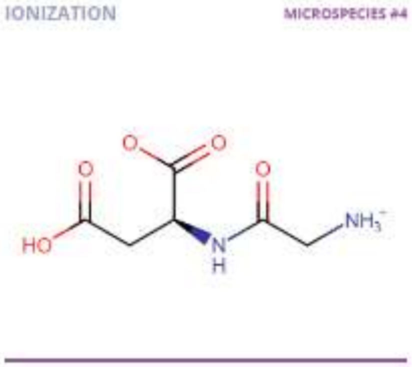<br>0.2%	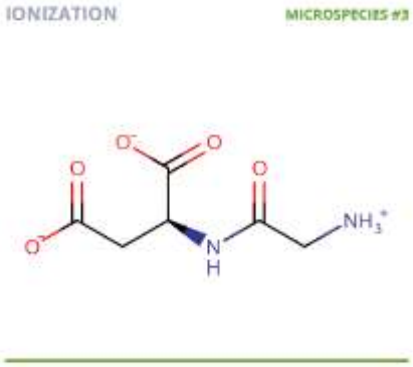<br>77.4%	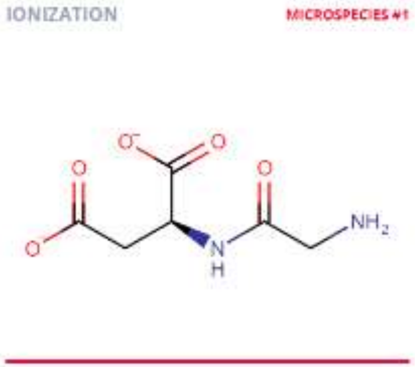<br>22.3%

	pH	+2	+1	0	-1	-2
**Asp-Gly**	**6.5**	**-**	**-**	**-**	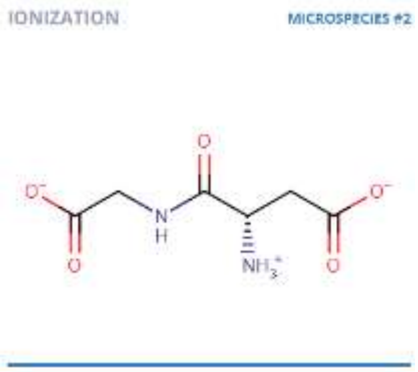 **98.9%**	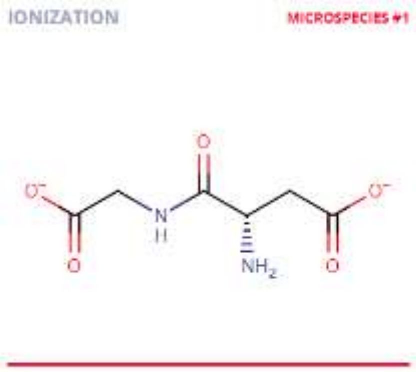 **1.0%**
	**7.6**	**-**	**-**	**-**	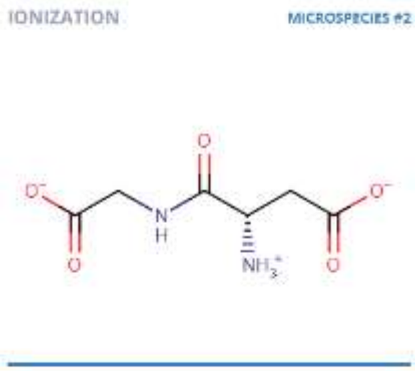 **89.4%**	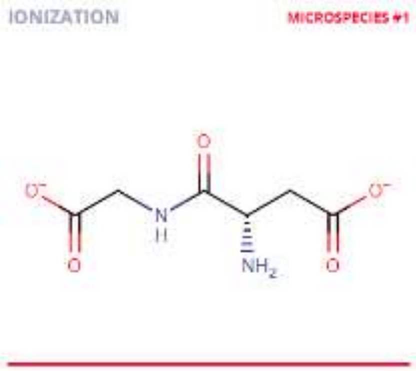 **10.6%**

(Statistical Summary Documents)

Supplemental Table 2

(please see file: Tab. 2S_ref_Fig. 2.pdf)

Supplemental Table 3

(please see file: Tab. 3S_ref_Fig. 3.pdf)

Supplemental Table 4

(please see file: Tab. 4S_ref_Fig. 4.pdf)

Citations

Berman H, Henrick K, Nakamura H. Announcing the worldwide Protein Data Bank. Nat Struct Biol. 2003 Dec;10(12):980. doi: 10.1038/nsb1203-980. PMID: 14634627.

Bienert S, Waterhouse A, de Beer TA, Tauriello G, Studer G, Bordoli L, Schwede T. The SWISS-MODEL Repository-new features and functionality. Nucleic Acids Res. 2017 Jan 4;45(D1):D313-D319. doi: 10.1093/nar/gkw1132. Epub 2016 Nov 29. PMID: 27899672; PMCID: PMC5210589.

Eastman P, Swails J, Chodera JD, McGibbon RT, Zhao Y, Beauchamp KA, Wang LP, Simmonett AC, Harrigan MP, Stern CD, Wiewiora RP, Brooks BR, Pande VS. OpenMM 7: Rapid development of high-performance algorithms for molecular dynamics. PLoS Comput Biol. 2017 Jul 26;13(7):e1005659. doi: 10.1371/journal.pcbi.1005659. PMID: 28746339; PMCID: PMC5549999.

Eberhardt J, Santos-Martins D, Tillack AF, Forli S. AutoDock Vina 1.2.0: New Docking Methods, Expanded Force Field, and Python Bindings. J Chem Inf Model. 2021 Aug 23;61(8):3891-3898. doi: 10.1021/acs.jcim.1c00203. Epub 2021 Jul 19. PMID: 34278794.

Killer M, Wald J, Pieprzyk J, Marlovits TC, Löw C. Structural snapshots of human PepT1 and PepT2 reveal mechanistic insights into substrate and drug transport across epithelial membranes. Sci Adv. 2021 Nov 5;7(45):eabk3259. doi: 10.1126/sciadv.abk3259. Epub 2021 Nov 3. PMID: 34730990; PMCID: PMC8565842.

Kim S, Chen J, Cheng T, Gindulyte A, He J, He S, Li Q, Shoemaker BA, Thiessen PA, Yu B, Zaslavsky L, Zhang J, Bolton EE. PubChem 2023 update. Nucleic Acids Res. 2023 Jan 6;51(D1):D1373-D1380. doi:10.1093/nar/gkac956.

Morris GM, Huey R, Lindstrom W, Sanner MF, Belew RK, Goodsell DS, Olson AJ. AutoDock4 and AutoDockTools4: Automated docking with selective receptor flexibility. J Comput Chem. 2009 Dec;30(16):2785-91. doi: 10.1002/jcc.21256. PMID: 19399780; PMCID: PMC2760638.

O'Boyle NM, Banck M, James CA, Morley C, Vandermeersch T, Hutchison GR. Open Babel: An open chemical toolbox. J Cheminform. 2011 Oct 7;3:33. doi: 10.1186/1758-2946-3-33. PMID: 21982300; PMCID: PMC3198950.

Pettersen EF, Goddard TD, Huang CC, Meng EC, Couch GS, Croll TI, Morris JH, Ferrin TE. UCSF ChimeraX: Structure visualization for researchers, educators, and developers. Protein Sci. 2021 Jan;30(1):70-82. doi: 10.1002/pro.3943. Epub 2020 Oct 22. PMID: 32881101; PMCID: PMC7737788.

Rizvi SM, Shakil S, Haneef M. A simple click by click protocol to perform docking: AutoDock 4.2 made easy for non-bioinformaticians. EXCLI J. 2013 Sep 23;12:831-57. PMID: 26648810; PMCID: PMC4669947.

Schrödinger L, DeLano W. PyMOL [Internet]. 2020. Available from: http://www.pymol.org/pymol.

Seeliger D, de Groot BL. Ligand docking and binding site analysis with PyMOL and Autodock/Vina. J Comput Aided Mol Des. 2010 May;24(5):417-22. doi: 10.1007/s10822-010-9352-6. Epub 2010 Apr 17. PMID: 20401516; PMCID: PMC2881210.

Trott O, Olson AJ. AutoDock Vina: improving the speed and accuracy of docking with a new scoring function, efficient optimization, and multithreading. J Comput Chem. 2010 Jan 30;31(2):455-61. doi: 10.1002/jcc.21334. PMID: 19499576; PMCID: PMC3041641.
